# Mucosal expression of *PI3*, *ANXA1*, and *VDR* discriminates Crohn’s disease from ulcerative colitis

**DOI:** 10.1038/s41598-023-45569-3

**Published:** 2023-10-27

**Authors:** Jaslin Pallikkunnath James, Boye Schnack Nielsen, Ib Jarle Christensen, Ebbe Langholz, Mikkel Malham, Tim Svenstrup Poulsen, Kim Holmstrøm, Lene Buhl Riis, Estrid Høgdall

**Affiliations:** 1 Department of Pathology, Herlev University Hospital, Borgmester Ib Juuls Vej 73, 2730 Herlev, Denmark; 2 Bioneer A/S, Hørsholm, Kogle Allé 2, 2970 Hørsholm, Denmark; 3 Gastroenheden D, Herlev University Hospital, 2730 Herlev, Denmark; 4 Institute for Clinical Medicine, University of Copenhagen, 2200 Copenhagen, Denmark; 5 The Department of Pediatric and Adolescence Medicine, Copenhagen University Hospital-Amager and Hvidovre, 2650 Hvidovre, Denmark; 6 Copenhagen Center for Inflammatory Bowel Disease in Children, Adolescents and Adults, Hvidovre Hospital, University of Copenhagen, 2650 Hvidovre, Denmark

**Keywords:** Transcriptomics, Gene expression analysis, Genetics, Molecular biology, Biomarkers, Diseases, Gastroenterology, Medical research, Molecular medicine, Pathogenesis

## Abstract

Differential diagnosis of inflammatory bowel disease (IBD) to Crohn’s disease (CD) or ulcerative colitis (UC) is crucial for treatment decision making. With the aim of generating a clinically applicable molecular-based tool to classify IBD patients, we assessed whole transcriptome analysis on endoscopy samples. A total of 408 patient samples were included covering both internal and external samples cohorts. Whole transcriptome analysis was performed on an internal cohort of FFPE IBD samples (CD, *n* = 16 and UC, *n* = 17). The 100 most significantly differentially expressed genes (DEG) were tested in two external cohorts. Ten of the DEG were further processed by functional enrichment analysis from which seven were found to show consistent significant performance in discriminating CD from UC: *PI3, ANXA1, VDR, MTCL1, SH3PXD2A-AS1, CLCF1, and CD180*. Differential expression of *PI3*, *ANXA1*, and *VDR* was reproduced by RT-qPCR, which was performed on an independent sample cohort of 97 patient samples (CD, *n* = 44 and UC, *n* = 53). Gene expression levels of the three-gene profile, resulted in an area under the curve of 0.84 (*P* = 0.02) in discriminating CD from UC, and therefore appear as an attractive molecular-based diagnostic tool for clinicians to distinguish CD from UC.

## Introduction

Inflammatory bowel disease (IBD) includes a group of chronic conditions that trigger inflammation in the lining of the gastrointestinal (GI) tract and affects more than 5 million people worldwide^1^. The diagnosis of IBD is based on clinical, endoscopic, and radiological characteristics together with histological evidence of structural and inflammatory changes^2^. The exact etiology of IBD remains unknown, however, it is believed to be caused by a mixture of genetic predisposition, abnormal immunity, and environmental exposures^3^. Crohn’s disease (CD) and Ulcerative colitis (UC) are the most common forms of IBD. UC is characterized by relapsing and remitting mucosal inflammation stretching from the rectum to proximal segments of the colon, whereas CD may affect the whole GI tract and cause transmural inflammation^4,5^. In CD, one-third of the patients have a pure colonic disease location and at least 60% of them have non-stricturing or non-penetrating behavior at the time of diagnosis. In the absence of granulomas, these cases can be difficult to differentiate from UC^4^, and are termed IBD unclassified (IBDU). Even though 25% of IBDU patients are eventually sub-classified into either CD or UC within 6 months of follow up, the majority remain as IBDU^4^ even after subsequent colectomy^6^. When confirming the subtype is crucial before surgery or when choosing an advanced medical treatment, it is necessary to separate IBDU cases into CD and UC at an early stage of the disease or during the disease. This underlines the need for biomarkers to support correct diagnosis of uncertain IBD cases for better disease management of the patients.

Since surgery, modern treatments, and emerging prognostic indices work on disease-specific strategies, it is important to determine the correct diagnosis from start for optimal clinical management in IBD patients. At present, there are several molecular diagnostic studies available for IBD, each offering valuable insights into disease mechanisms and patient management, however there are so far no universal molecular diagnostic biomarkers . Serologic tests and fecal markers have been studied intensively to improve the diagnosis of IBD. Fecal markers such as lactoferrin and calprotectin are indicative for any inflammation in the GI tract, which can discriminate between IBD and functional disorders without inflammation but have proved unsuccessful in distinguishing CD from UC. Serologic markers such as autoantibodies, and neutrophils^7–9^ are not alone sufficient to have a distinctive diagnosis in IBD. There have been growing efforts to characterize and explore the pathogenic mechanisms within the inflammatory cascade of IBD subtypes to identify more appropriate treatment regimens on a case-by-case basis. By using differential gene expression of 7 marker genes on frozen biopsies, Stein et. al developed a biomarker tool for differential diagnosis in IBD, thereby demonstrating that CD and UC have distinct expression signatures^10^. Former studies have showed that IBD subtyping using fresh frozen colon biopsies can be performed, but the results have not been substantiated in follow-up studies^11–13^. Formalin-fixed, paraffin-embedded (FFPE) colon tissue biopsies may have significant promise for analyzing IBD, IBDU, and several other diseases because they are representative tissues collected and preserved by pathologists worldwide. FFPE tissues are routinely produced in the clinical routine setting and can be stored at room temperature for decades. One of the concerns when using FFPE tissue samples is the risk that the mRNAs may be of poor quality due to cross-linking or oxidative deamination which hampers subsequent gene expression studies. However, Turnbull et al. showed that comparable gene expression profiles can be obtained using FFPE material from breast tumor samples and FFPE is a reliable substitute to fresh frozen biopsies^14^. Importantly, also using FFPE colon biopsy samples, Christophi et. al quantified gene expression by RT-PCR, which gave insights into the pathogenetic differences between CD and UC^15^.

In this study we aimed at identifying biomarkers for discriminating CD from UC patients using FFPE-derived RNA from archived patient samples.

## Results

### Discovery study using next generation sequencing

**Figure 1:**
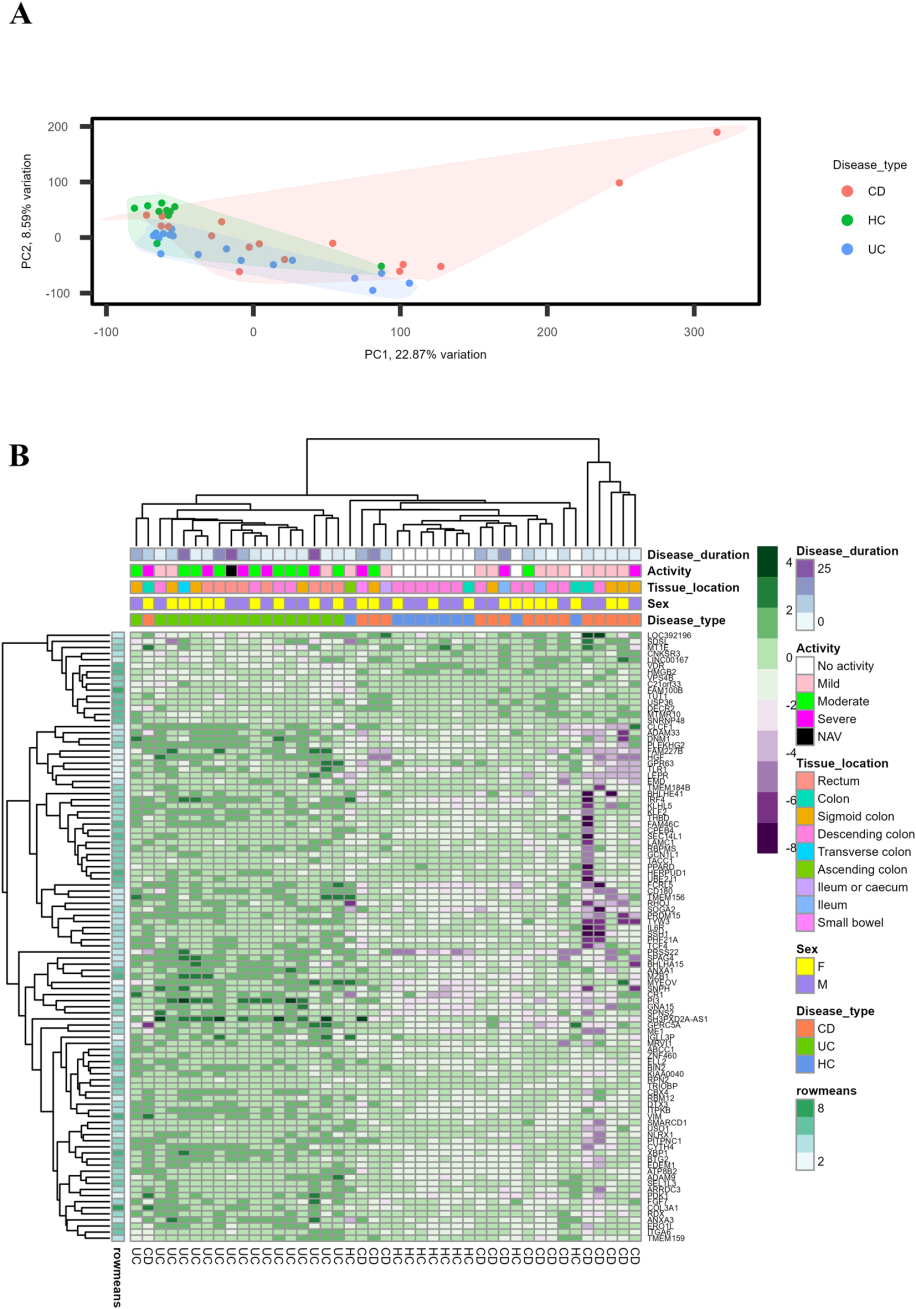
Heatmap and unsupervised hierarchical clustering of differentially expressed genes between CD (16) and UC (17) samples from the discovery study. (**A**) Principal component analysis (PCA) plot showing the unsupervised clustering of the discovery study samples into CDs, UCs, and healthy controls (HC) with the normalized gene expression values. Each point represents one sample with colors indicating which group as indicated in the figure. (**B**) Heatmap of the best 100 differentially expressed genes based on the *P* values from the discovery study. Factors such as disease duration, disease activity, tissue location, sex and disease type are shown with colored bars at the top of the heatmap. Disease duration is presented in years from 0 to 25 years. Purple color represents the highest expression and green color being the lowest expression for corresponding genes in respective samples. *PC* principal component, *CD* Crohn’s disease, *UC* ulcerative colitis, *HC* healthy control, *NAV* not available, *F* female, *M* male.

For the discovery study, total RNA was extracted from mucosal FFPE tissue sections from 33 IBD and 10 HC cases with a mean RNA concentration of 45.19 ng/µL (range: 7 to 156 ng/µL). Targeted RNA sequencing with the Ion AmpliSeq™ transcriptome human gene expression panel resulted in an average of 13 million (range: 2 to 32 million) mapped reads per sample. The average fraction of valid reads among all the mapped reads per sample was 83% (range, 48 to 95%) and that of the targets detected in the valid reads were with an average of 65% (range, 36 to 75%) per sample. Unsupervised clustering of the samples based on gene expression using the PCA plot, clustered the samples into different disease types as shown in Fig. 1A. Out of more than 20,800 genes in the transcriptome panel, 18,848 genes were selected for further analysis and is uploaded into GEO database (GSE222070). Genes with less than 10 reads in all samples were excluded, which resulted in a list of 14,190 genes. From the logistic regression analysis, we found that a set of 429 genes were significantly differentially expressed between CD and UC with 85 genes being up-regulated and 344 genes being down-regulated in CD relative to UC. Hierarchical clustering of differentially regulated genes separated CD and UC as shown in the heatmap, Fig. 1B.

### Testing in external cohorts

The set of 100 most significantly expressed genes from our cohort (fold change, median = 2.48, IQR 1.95–3.2) was tested in two external cohorts from GEO database having gene expression data from mucosal biopsies of CD and UC patients: GSE16879^16^ and GSE117993^17^. Both GSE16879^16^ and GSE117993^17^ are based on RNA from frozen samples, but obtained the expression data with different sequencing methods, Affymetrix micro-array and Illumina HiSeq 2500 sequencing platforms, respectively. OR and AUC values for all the 100 genes in the three different datasets are shown in supplementary Table 2. Among these, *PI3* showed the highest AUC in all three datasets [0.9044 (OR, 0.2137), 0.8271 (OR, 0.4223), and 0.7939 (OR, 0.5650) in GSE222070 (1: Herlev), GSE117993^17^ (2: GSE11799), and GSE16879^16^ (3: GSE16879), respectively]. This confirmed that our results are robust and reproducible also in cohorts of different ethnicities, type of sample material, and sequencing methods.

### Functional enrichment analysis

**Figure 2:**
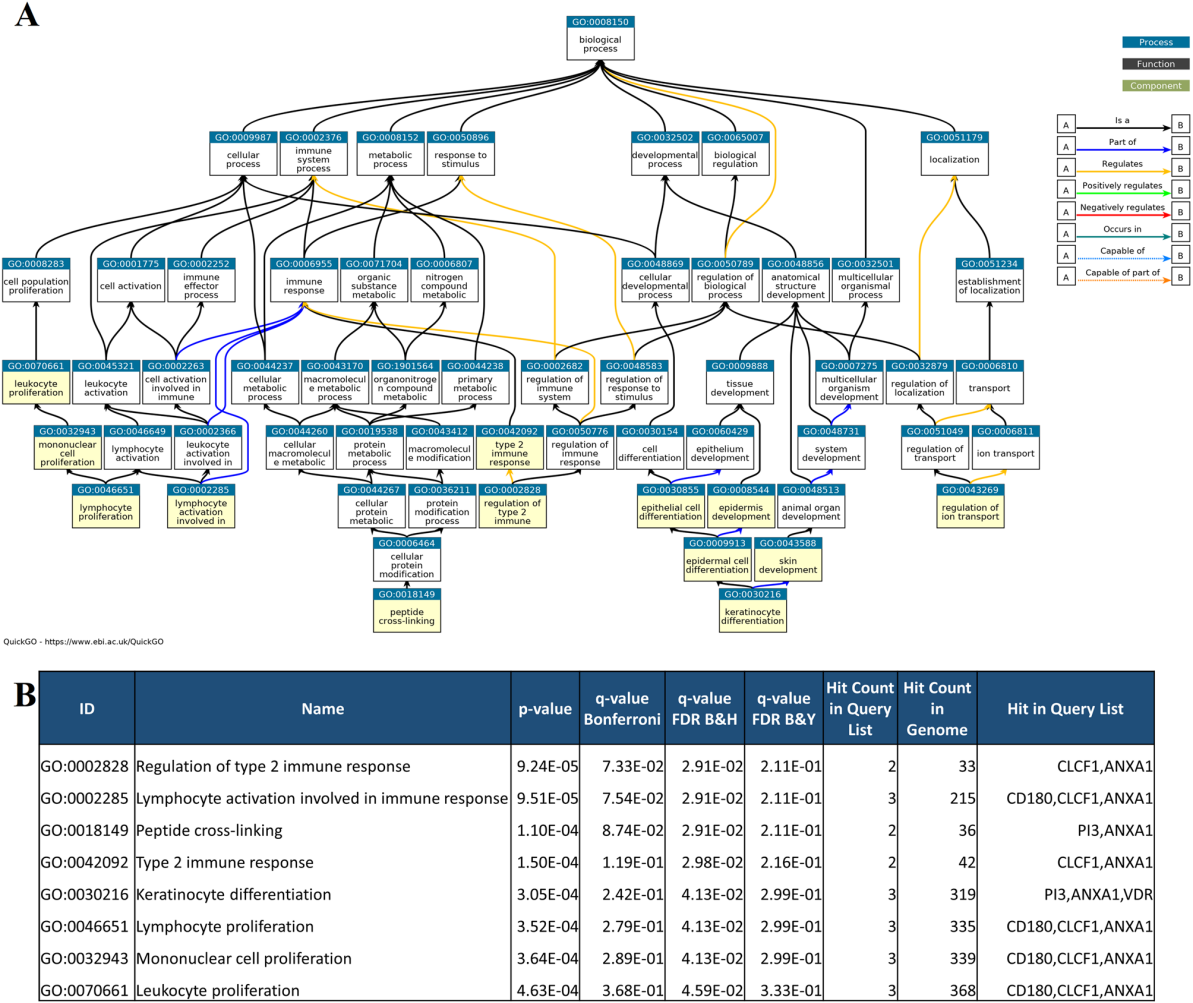
Functional enrichment analysis of the differentially expressed genes. Differentially expressed genes between CDs and UCs identified in the discovery study after testing in two external cohorts and enriched biological processes associated with them: *PI3*, *ANXA1*, *VDR*, *MTCL1*, *SH3PXD2A*-*AS1*, *CLCF1*, *ME1*, *RPBMS*, *PRSS22,* and *CD180*. (**A**) Flow chart of the enriched biological processes with the 10 selected genes as highlighted in yellow. (**B**) Gene ontology (GO) terms with *P* values and the corresponding genes involved in the respective biological processes. Functional enrichment analysis was performed using ToppGene and QuickGO.

**Figure 3:**
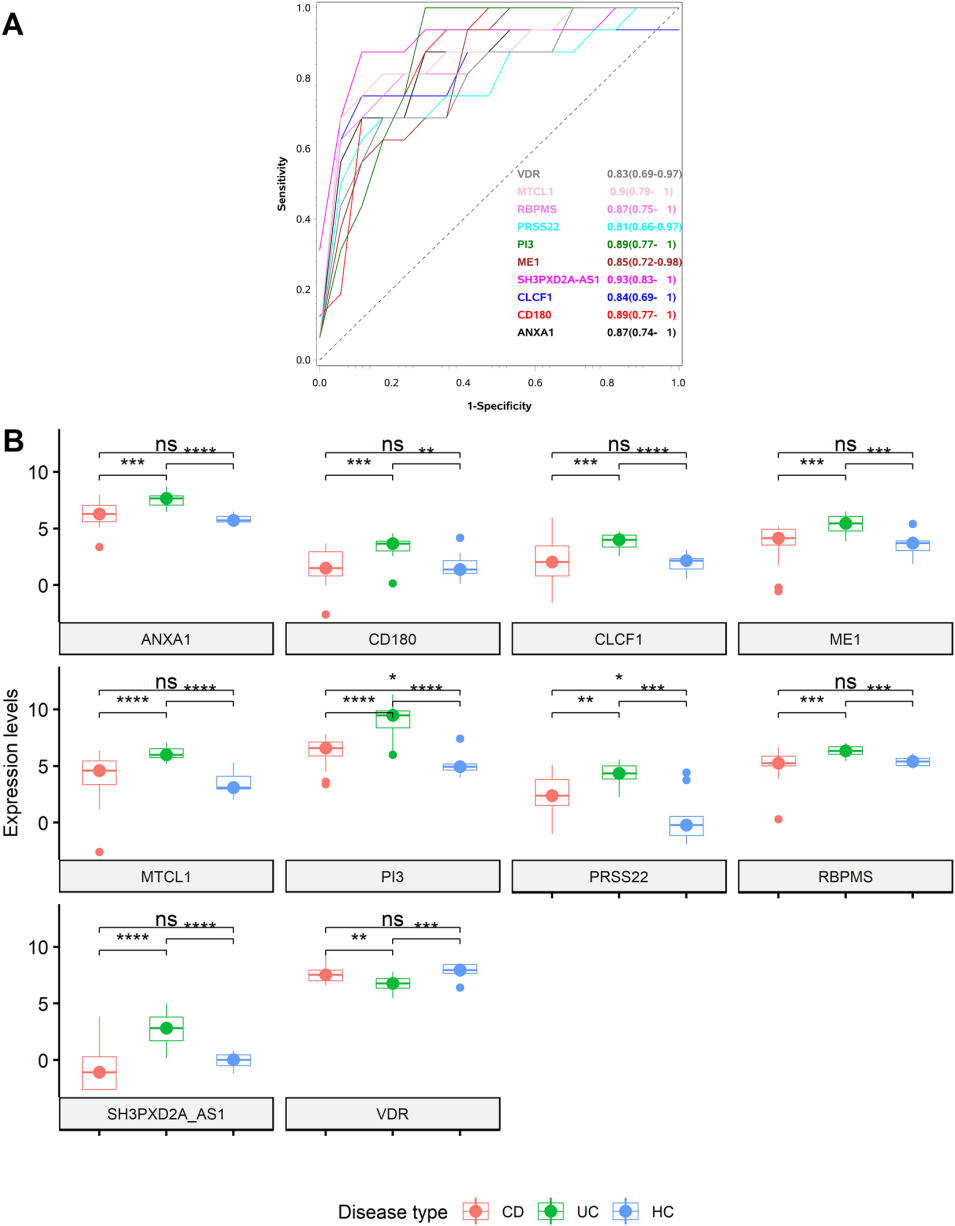
Expression levels of the differentially expressed genes *PI3*, *ANXA1*, *VDR*, *MTCL1*, *SH3PXD2A*-*AS1*, *CLCF1*, *ME1*, *RPBMS*, *PRSS22*, and *CD180* in CD, UC and HC based on sequencing assay on the GSE222070 (discovery cohort). (**A**) ROC plot with AUC values (confidence intervals) for the corresponding genes in discriminating CD (16) from UC (17) samples among the discovery study samples. (**B**) Box plot of the 10 gene candidates and their expression levels in CD, UC, and HC samples from the discovery cohort. Symbols "****", "***", "**", "*" and "ns" corresponds to *P* values < 0.001, 0.001, 0.01, 0.49 and ≥ 0.05 respectively.

Based on AUC values in three datasets, expression of a set of ten genes, *PI3*, *ANXA1*, *VDR*, *MTCL1*, *SH3PXD2A*-*AS1*, *CLCF1*, *ME1*, *RPBMS*, *PRSS22* and *CD180*, was selected for enrichment analysis. These differentially expressed genes (DEG) were subjected to functional enrichment analysis and the results are shown in Fig. 2A ^18,19^. The DEGs were typically involved in biological processes such as proliferation, immune response, tissue development and ion transport. With the analysis of cellular components, DEGs were mainly enriched in extracellular matrix and plasma membrane. Molecular functions such as protease binding, single stranded RNA binding and serine hydrolase activities were enriched. Receiver operating characteristic statistics discriminating CD from UC in the discovery study cohort for the 10 selected genes are shown in Fig. 3A. Box plots comparing expression levels of the 10 most relevant candidates among CD, UC, and HC are shown in Fig. 3B. A panel of 7 genes (*PI3*, *ANXA1*, *VDR*, *MTCL1*, *SH3PXD2A*-*AS1*, *CLCF1*, and *CD180*) out of the initially 10 genes were selected for further validation based on their relation to the extra intestinal manifestations (EIM) connected to IBD and involvement in immune response. Other factors considered in the selection process were high expression levels, allowing detection with basic methods such as RT-qPCR or ISH, and previous literature^20–28^. Expression of *PI3*, *ANXA1*, *VDR*, *MTCL1*, *SH3PXD2A*-*AS1*, *CLCF1*, and *CD180* have been shown to be involved in immune response, lymphocyte proliferation and keratinocyte differentiation. E.g. the protein encoded by *PI3*, Elafin, in serum is known as a marker for psoriasis and inflammation^29^, and *ANXA1* is highly expressed in intestinal tissue of UC patients compared to CD patients and promotes mucosal homeostasis^24^, see also Fig. 2B, and the discussion section below. Complete list of gene ontology analysis results is presented in supplementary file 4.

### Validation study using RT-qPCR

**Figure 4:**
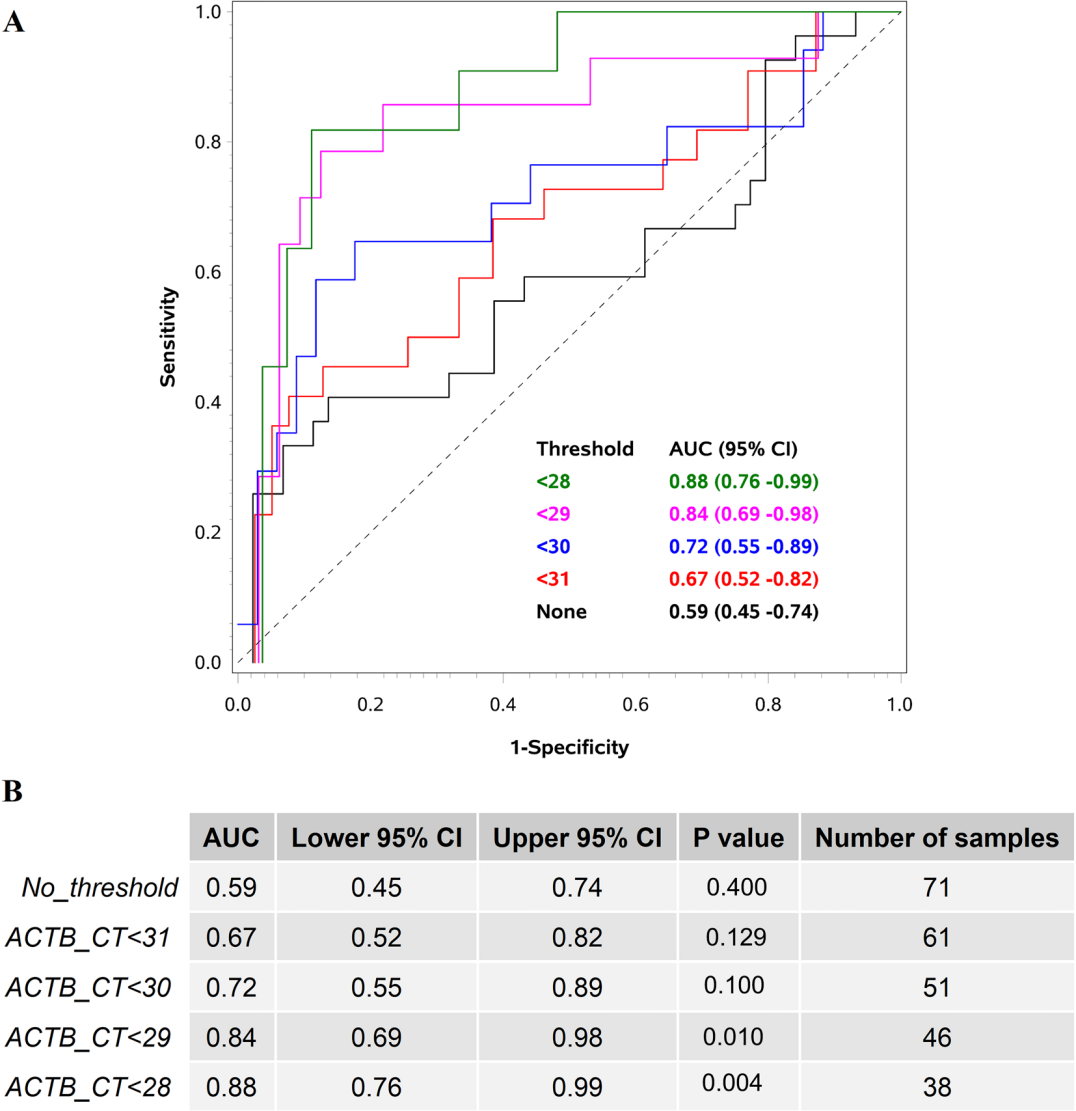
ROC plot showing the predictive power of the algorithm in the validation cohort. (**A**) ROC plot with AUC values for discriminating Crohn’s disease (CD) and ulcerative colitis (UC) based on the algorithm using RT-qPCR expression values of PI3*,* ANXA1*,* and VDR mRNAs in combination with the age and sex of the patient at different endogenous control thresholds. ACTB mRNA was used as the endogenous control for the RT-qPCR assay. This data is generated based on the gene expression values from the validation cohort consisting of 44 CD and 53 UC patient samples. (**B**) Table with AUC values, *P* values, confidence intervals (CI) and number of samples included in the analysis at different ACTB mRNA threshold values.

The final set of seven selected genes were tested in an independent cohort of 109 FFPE mucosal biopsies including 45 CDs, 53 UCs, and 12 HCs using RT-qPCR method. Expression of β-actin mRNA (*ACTB*) was used as the endogenous control for the assay and was found to be a key factor for the analysis, thus, the highest AUC values were obtained when samples with poor *ACTB* Ct values (> 28) were removed. RT-qPCR expression values of *PI3*, *ANXA1,* and *VDR* along with the age at diagnosis and sex of the patients resulted in an AUC of 0.84 (*P* = 0.02) in discriminating CD from UC (Fig. 4A). Higher expression of *VDR* alone was detected in CD samples with an AUC of 0.77 (*P* = 0.01). The Ct values for ACTB varied from 28 to 31 among the samples, which indicated that efficiency of the discriminating algorithm was improved with increasing quality of RNA (Fig. 4B). Consequently, the number of samples included in the analysis was also reduced from 61 samples at ACTB threshold < 31 to 38 samples at ACTB threshold < 28, see also Fig. 4B. With an ACTB threshold < 31 the AUC for discriminating CD from UC was 0.67 (*P* = 0.61) whereas an ACTB threshold < 28, increased AUC to 0.88 (*P* = 0.001). Expression of *MTCL1*, *SH3PXD2A*-*AS1*, *CLCF1,* and *CD180* were found to be below the detection limit of the RT-qPCR method and excluded from the analysis.

### Localization of *PI3*, *ANXA1* and *VDR*

**Figure 5:**
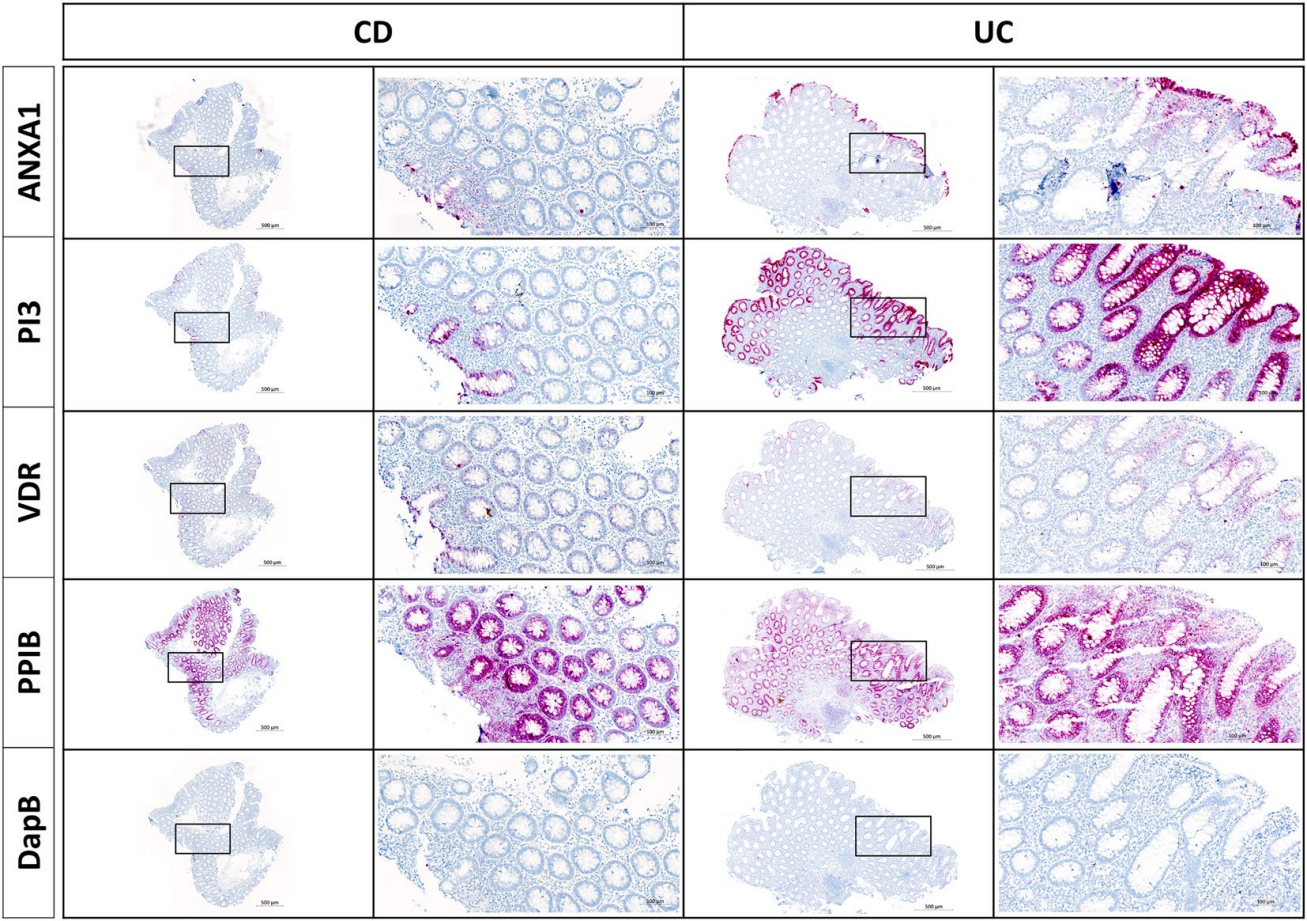
In situ hybridization (ISH) for ANXA1, PI3 and VDR mRNAs in CD and UC. RNAScope probes for the genes ANXA1, PI3 and VDR were applied to mucosal biopsies from a CD and a UC sample. The two samples are presented with overview images having framed areas shown in the zoomed images. UC samples in general showed relatively strong expression of *PI3* and *ANXA1* in the epithelial cells, compared to CD samples which in general had low expression of *PI3* and *ANXA1*. Conversely, *VDR* was intensely expressed in CD samples compared to UC samples. PPIB and DapB mRNA probes were used as positive and negative controls, respectively. The PPIB mRNA ISH signal is equally intense in the 2 samples. No ISH signal is seen with DapB probe.

A subset of 18 FFPE samples from the discovery study were selected for localization study based on the amount of remaining tissue and quality of the material. The samples were stained with RNAscope probes to PI3, ANXA1, and VDR mRNAs in parallel with PPIB mRNA and bacterial dapB mRNA probes as positive and negative controls, respectively (Fig. 5). UC samples showed relatively strong expression of *PI3* and *ANXA1* in the epithelial cells, whereas CD samples had low expression of *PI3* and *ANXA1*. VDR mRNA was intensely expressed in the epithelium of CD samples compared to UC samples. All three genes’ relative staining intensities were in concordance with the sequencing and RT-qPCR results. PI3 mRNA and ANXA1 mRNA were detected mainly in the mucosal epithelium, and with the strongest staining being located towards the lumen. There was no PI3 mRNA signal in the lymphoid follicles (LF) or lamina propria, whereas ANXA1 mRNA was also detected in lamina propria and in the LF. VDR mRNA was highly expressed in the epithelium, but only sporadic staining was noted in the LF.

## Discussion

**Figure 6:**
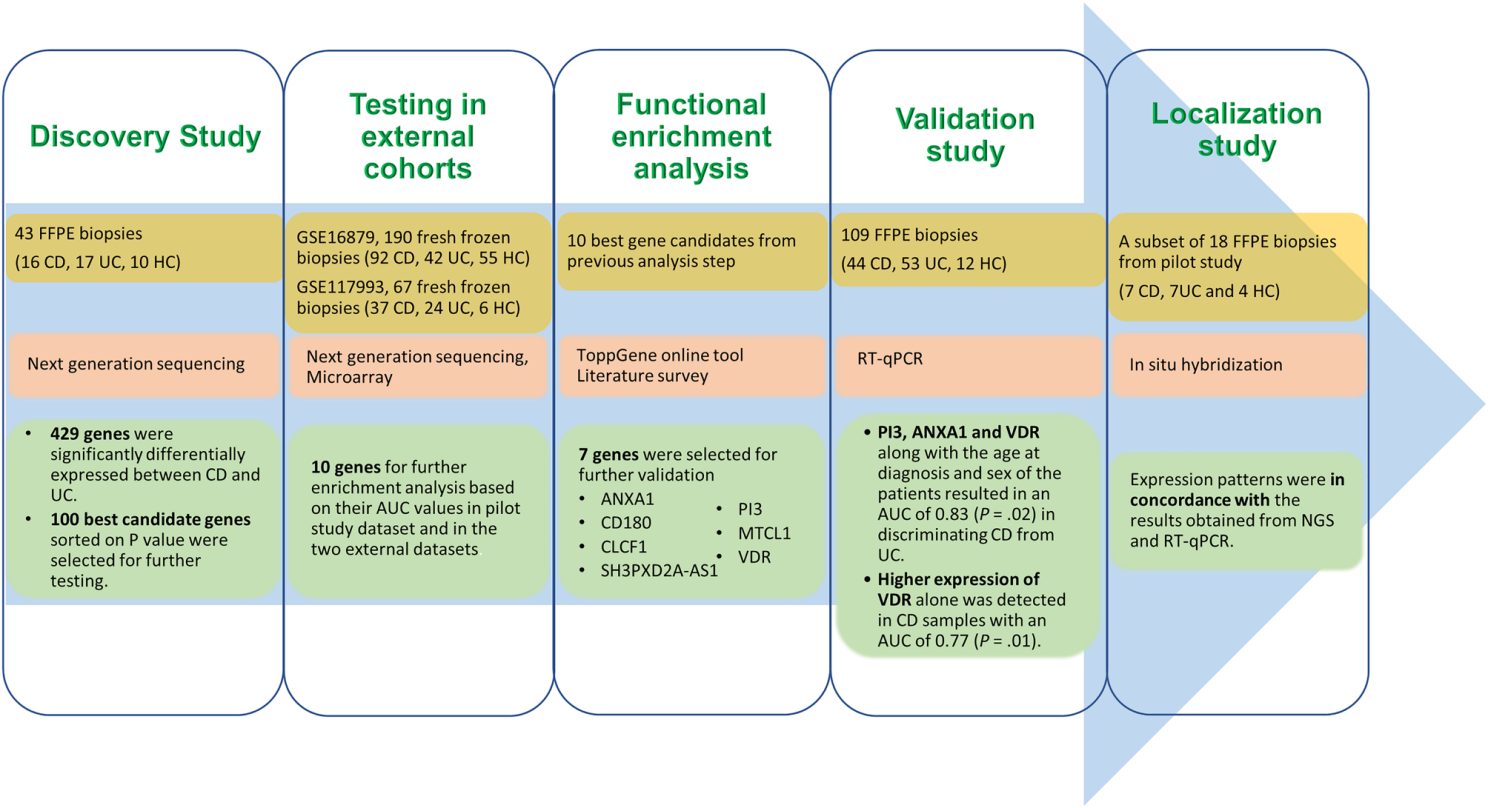
Schematic illustration of the study design and strategy. Summary of samples used, methods applied, and key results obtained are shown as a flow chart.

Using whole transcriptome sequencing on archived mucosal biopsies of IBD patients, we identified 429 genes differentially expressed between CD and UC. After testing in two external IBD cohorts, a list of the ten best candidate genes were selected for further validation. Functional enrichment analysis and validation using RT-qPCR in a third independent cohort, paved the way for an algorithm based on three genes (ANXA1, PI3 and VDR), which, in combination with patient’s age at diagnosis and sex, can discriminate CD from UC with an average precision of 84%. The workflow of the whole study and the key results are summarized in Fig. 6.

Based on the molecular profile, the presented algorithm has the potential to support IBD subtyping at disease presentation and thereby to plan the optimal treatment course. Earlier studies have reported that IBD molecular subtyping is possible with differential gene expression from fresh frozen colon biopsies and FFPE tissue biopsies^10,15,30,31^. Other studies have also used differential expression of microRNAs, lamina propria T-helper subsets, and matrix-assisted laser desorption/ionization (MALDI) imaging techniques aiming at discriminating CD and UC^32,33^. However, the earlier findings have not been extensively supported in follow-up studies^11–13^.

In this study, we obtained a sample cohort of 43 archived FFPE colon biopsies for the discovery study and another 109 archived FFPE colon biopsies for the RT-qPCR validation. The discovery study sample cohort consisted of a mix of samples with different clinical characteristics such as disease subtype, sex, level of inflammation, disease duration, treatment status, and site of the disease. To test the gene expression differences based on the age at diagnosis, one-third of our validation cohort were pediatric patients. Our proposed 3-gene algorithm was validated in external data sets comprising 134 and 61 fresh frozen rectal or colon biopsies including both adults and children^34,35^, thus, emphasizing the advantage of covering diversity in the selection criteria terms. We used total RNA extracted from the archived FFPE blocks with relatively low RNA integrity numbers (RIN) ≤ 2. Despite of this, our molecular profile was demonstrated to be robust since it was reproduced in external datasets that were based on different sample types and sequencing platforms, as well as by being verified in our own independent sample cohort using RT-qPCR and partly by ISH. We believe that RT-qPCR is a feasible and cost-efficient approach to perform the molecular-based diagnosis in the daily routine. Although the sample size of this retrospective cohort is limited, there is sufficient power to detect a number of highly significant differences between disease groups.

IBD is believed to be caused by reduced barrier function, and the mucous epithelial barriers play a vital role in regulating tissue homeostasis^36^. In normal conditions, the inflammatory response and tissue repair are precisely balanced to guarantee restoration of mucosal homeostasis^36^. In IBD, such resolution of inflammation is compromised and thereby leads to sustained inflammation and impaired healing^37^. Even though UC and CD both are associated with reduced barrier function, their etiology and treatment regimens are different. UC is characterized by acute and chronic inflammation in the colonic mucosa, whereas in CD the inflammation is transmural, and may be characterized by occurrence of complications such as stenosis and fistulas^6,38^. In our functional enrichment analysis, we found that the expression of *ANXA1, PI3,* and *VDR* are involved in tissue homeostasis and immune related biological processes. Whereas *ANXA1* and *PI3* were high in UC, *VDR* expression was high in CD. All three genes are well-known parameters in IBD and in the maintenance of epithelial barrier integrity.

In line with our findings, previous studies have also reported elevated expression levels of *ANXA1* in UC patient samples and loss of *ANXA1* expression in patient samples with progressive CD compared to healthy controls^25,39^. By ISH analysis we found ANXA1 mRNA in both epithelial cells and a subset of the cells located in lamina propria and submucosa. Similarly, ANXA1 protein (annexin-A1) has been observed in monocytes, macrophages, neutrophils, and epithelial cells^40^. Annexin-A1 is a phospholipid-binding protein that acts in a variety of inflammatory pathways^41^. In their recent review, Leoni and Nusrat^42^ discuss the repair-promoting functions of annexin-A1 within the inflamed mucosa^42^. Indeed, *ANXA1* deficient mice exhibited increased susceptibility to DSS-induced colitis^43^. Together, our findings support the hypothesis that annexin-A1 promotes the clearance of inflammation and restoration of compromised mucosal integrity and suggest that the high expression of *ANXA1* in the epithelium of UC samples serves as a defense mechanism.

Peptidase Inhibitor 3 (PI3) encodes the protein originally named SKALP or elafin, where elafin is formed by processing of the precursor protein trappin-2 by transglutaminases^44–46^. *PI3* is induced in keratinocytes by inflammatory mediators like tumor necrosis factor α^47^. Elafin functions as a protease inhibitor and inhibits elastase 2a (ELA2A), recently discovered to be highly expressed in IBD and suggested to drive inflammation and loss of barrier function^48^. Elafin is well-known in anti-inflammatory processes as a defense against luminal microbes by its ability to disrupting bacterial cell membranes and by suppressing inflammation-associated transcription factors like NF-kB and AP-1^49,50^. In addition to being expressed in epithelium, elafin has also been found in neutrophils, the main function being anti-inflammatory, anti-microbial and pro-resolution of inflammation^22^. In the murine DSS model, mice with transgenic over-expression of elafin, elafin protected against the development of colitis^51^. In the IBD cases included in our study, as well as in those studied in Arijs et al^16^, Haberman^17^, Flach et al^52^, the PI3 mRNA levels were higher in UC and CD patient mucosa than in HC. This finding was supported by our RT-qPCR and in situ hybridization analyses, showing abundant PI3 mRNA in the mucosal epithelium of UC patients. In addition, immunohistochemical analysis found elafin strongly enhanced in inflamed versus non-inflamed UC^53^ and elafin protein levels measured in serum were elevated in UC patients^23,47^, suggesting that the increased PI3 mRNA levels is reflected in increased elafin protein levels. In the UC tissue, the increased level of PI3/elafin expression however appears insufficient to impede the hostile inflammation in the mucosa, and it is therefore encouraging that experimental therapeutic approaches using elafin have proven promising^54^.

In contrast to *ANXA1* and *PI3*, we found elevated *VDR* expression in CD compared to UC. Vitamin D/VDR signaling directs homeostasis, stimulates functionality of tight junction proteins and is important for bacteria recognition, pathogen clearance and dysbiosis prevention^55^. Cellular response to vitamin D was among the top hits of negatively enriched GO terms in non-inflamed CD patients compared to healthy controls in a study with treatment-naive IBD patients and their mucosal gene expression, thus, strengthening the body of evidence regarding vitamin D as a significant actor in the pathogenesis of CD^56,57^. The active form of vitamin D, calcitriol, achieves its effects through *VDR*, which functions as a transcription factor in the cell nucleus and controls gene expression. *VDR* regulation of e.g. Claudin-15 is important in maintaining epithelial integrity and for protecting against colitis^38,58^. *VDR* appears to have several functions as transcription factor and the low *VDR* expression in IBD has been discussed to correlate with dysfunctional vitamin D/VDR signaling in IBD patients^59^.

Autoimmune inflammatory conditions are common for IBD and psoriasis, and it may therefore not seem surprising that “keratinocyte differentiation” showed up in the functional enrichment analysis. Interaction between keratinocytes, immune cells, and other skin-resident cells is involved in the pathogenesis of psoriasis^60^. E.g. studies have shown that elafin is upregulated in psoriasis^29,61,62^, similar to our finding of *PI3* upregulation in UC. From the functional enrichment analysis, we found that also *ANXA1* and *VDR* are involved in keratinocyte differentiation and tissue development. Previous studies have shown that CD and psoriasis are related within individuals, leading to relatively more CD patients also suffer from psoriasis, and also that the level of association of psoriasis to CD is higher than to UC^63^.

It is noted that the quality of RNA from FFPE samples can be a challenge. Increasing the quality of RNA could give higher discriminating power for the RT-qPCR method, see also Fig. 4B. If implemented in routine practice, the quality of RNA is likely to be higher, as the FFPE samples will not be stored for long time. To avoid gender-related bias or prejudice based on other clinical characteristics including the level of inflammation, treatment history, or illness location, samples were chosen using a strict and unbiased random sampling procedure. Despite our best efforts to create a completely balanced gender distribution, for instance, a slight variation of 3–9% more female patient samples—which we estimate is within the range of normal statistical fluctuation—occurred.

Since the samples we utilized were drawn at random, they came from both the time of the diagnosis and later in the course of the treatment. Another limitation may be that our samples were selected with a clear CD or UC phenotype and not cases with an uncertain phenotype. This precludes the possibility of testing the algorithm in IBDU cases.

In conclusion, we have developed an algorithm based on RT-qPCR expression values of 3 genes in combination with the patient's age and sex, to support clinicians in distinguishing CD from UC. This method could be applied in a molecular pathology routine clinical setup and potentially to categorize IBDU cases into CD and UC patients at early presentation of disease or during disease course when reconfirmation of the subtype is of outmost importance prior to surgery or choice of advanced medical treatment.

## Methods

### Samples

FFPE mucosal tissue biopsies were used for the discovery study [33 IBD patients and 10 healthy controls (HC)] and the validation study (97 IBD patients and 12 HCs). The samples were stored in clinical biobanks at hospitals in the capital region of Denmark as part of the initial diagnostic procedure. Patients were selected according to IBD subtype and had diverse disease characteristics regarding age at diagnosis, degree of inflammation, treatment history, and disease location. Patient characteristics are presented in Table 1. According to the amount of tissue remaining in the FFPE block, 18 samples (7 CDs, 7UCs and 4 HCs) were selected from the discovery study cohort with varying sample characteristics for localization study using in situ hybridization (ISH).

**Table 1:** Patient characteristics of the discovery study and validation study cohort.

	Discovery Study cohort (N = 43)			Validation study cohort (N = 109)		
	CD	UC	HC	CD	UC	HC
Number of samples	16	17	10	44	53	12
Age at diagnosis						
Median (IQR)	27 (24–46)	34 (29–43)	NAP	25 (14–45)	24 (15–37)	NAP
Sex						
Female, N (%)	9 (56)	9 (53)	5 (50)	26 (59)	28 (53)	5 (42)
Age of the FFPE sample blocks in years						
Median (range)	4 (0–11)	1 (0–9)	0 (0–9)	6 (1–20)	5 (1–15)	1 (1–9)
Rate of inflammation						
Mild/moderate/severe (N)	10/2/4	3/9/4*	1**/0/0	20/10/14	21/25/7	NAP
Disease duration in years						
Median (range)	4 (0–19)	1 (0–27)	NAP	2 (0–30)	1 (0–20)	NAP
Medical treatment (non-biological) at time of biopsy						
Yes/no/NA(N)	10/6/0	13/3/1	1/0/0	42/0/2	46/0/7	NAP
Medical treatment (biological) at time of biopsy						
Yes/no/NA (N)	2/14/0	3/13/1	NAP	17/25/2	14/32/7	NAP
Number of samples and sample location						
Small bowel	1					
Colon (unspecified)	2		2	10	9	1
Ileum/cecum	3			14		
Ascending colon			1	4	4	1
Transverse colon		1		2		
Descending colon	5	4	7	2	10	5
Sigmoid colon	5	4		7	19	5
Rectum		8		5	11	

*N* number of samples, *CD* Crohn’s disease, *UC* ulcerative colitis, *HC* healthy controls, *NAP* not applicable, *IQR* inter quartile range, *FFPE* formalin fixed paraffin embedded, *NA* not available.

*One sample information is not available.

**Slight inflammation due to bowel preparation prior to endoscopy.

### RNA extraction

Total RNA was isolated from two 10 µm thick sections of FFPE tissue samples using MagMAX™ FFPE DNA/RNA Ultra Kit from Applied Biosystems™ according to the manufacturer’s instructions. RNA quantity and purity were assessed using the NanoDrop spectrophotometer (ThermoFisher Scientific) and Qubit RNA HS Assay kit (Life Technologies). RNA quality and integrity were evaluated using Agilent RNA 6000 Nano Kit on Agilent 2100 Bioanalyzer System. RNA samples were kept at − 80 °C until further use. All RNA samples used for analysis had an RNA integrity number value of less than 2.

### Next generation sequencing

Total RNAs were diluted to 10 ng using nuclease free water and reverse transcribed using SuperScript™ VILO™ cDNA Synthesis Kit. Transcriptome libraries were generated using automated library preparation with the Ion AmpliSeq™ Transcriptome Human Gene Expression Panel containing 20,800 primer pairs and the Chef-Ready Kit (A31446). Transcriptome libraries were barcoded, templated, and sequenced on Ion GeneStudio™ S5 System using the Ion 550 chip. Initial analysis for AmpliSeq sequencing data was performed using the ampliSeqRNA plugin (V5.12) available with Ion Torrent™ sequencing platforms using Torrent Mapping Alignment Program (TMAP). Raw sequencing reads were aligned against the reference library hg19 Ampliseq transcriptome V1.1. Raw read counts obtained from Ampliseq were used for further statistical analysis.

### Statistical analysis of NGS data

Raw read counts obtained from ampliSeqRNA plugin were filtered, normalized using the trimmed mean method^64^ and log transformed in R using the packages limma and edgeR^65^. Statistical calculations were based on logistic regression comparing the expression results of samples obtained from patients with CD and UC diagnoses. The probability of CD was modeled. Multivariate analysis on the factors such as selected genes, age, sex, and disease duration were performed. Model assessment has been done using goodness of fit (Hosmer–Lemeshow) statistics. The best 100 genes were selected based on the p-values conditional on the numerical value of the regression coefficient be greater than one i.e., the odds ratio was greater than 2.71. Results are presented by odds ratios (OR) with 95% confidence limits, the AUC [area under the receiver operating characteristic curve (ROC)] as a measure of discrimination, and the p-value. Expression levels for HC samples were compared to CD and UC using T-test. P-values less than 5% were considered significant. Analysis was performed using the R^66^ packages limma^67^, edgeR^68^ and SAS (v9.4, SAS Institute, Cary, NC., USA).

### Testing in external cohorts

Gene expression omnibus (GEO) were searched for datasets containing references to IBD and related studies. Datasets GSE16879^16^ and GSE117993^17^ were chosen based on the sample size of the cohort, type of samples and type of techniques used. Fresh frozen samples were used in both GEO datasets. Both external datasets have used other platforms (Illumina HiSeq 2500 and Affymetrix Human Genome U133 Plus 2.0 Array) for the gene expression and different method for normalization analysis (moderated T-statistics). The 100 best performing genes from our cohort were assessed in the external datasets by logistic regression. Comparisons between CD and UC were only performed, not with HC. Due to differences in platforms used for RNA sequencing, some of the genes in Ion AmpliSeq™ Transcriptome Human Gene Expression Panel was having different nomenclatures in the external datasets. Two of the genes were missing in both external datasets and one gene was present only in one of the datasets.

### Functional enrichment analysis

Genes having an AUC of greater than 0.70 in all three datasets were selected for further functional enrichment analysis. Functional annotation enrichment analyses of the ten most significant genes were performed using ToppFun function in ToppGene online tool (https://toppgene.cchmc.org/). Related genes were grouped according to features such as gene ontology (GO): molecular function, biological processes, and cellular component, and disease. FDR B&H cutoff was set to 0.05. Depending on the molecular functions, previous literature in relation to IBD and level of expression in colonic mucosa, 7 genes were selected for further validation using RT-qPCR.

### RT-qPCR

Expression of the 7 genes panel were tested in a validation cohort of 109 FFPE samples having 44 CDs, 53 UCs and 12 HCs using RT-qPCR. Total RNA from the FFPE mucosal tissue sections were extracted using MagMAX™ FFPE DNA/RNA Ultra Kit from Applied Biosystems™ and were quantified using Quant-iT™ RiboGreen RNA reagent and kit from Invitrogen. RT-qPCR was performed using QuantiNova SYBR^®^ Green RT-qPCR Kit (Qiagen). QuantiNova LNA PCR Custom Panel (GeneGlobe ID: SBCA09807) was designed with the 7 selected genes. The primers used in the custom panel are listed in supplementary file 1. HS_PPC_2467741, HS_HGDC_2467744 and HS_QIC_2467742 were used as reference assays according to custom panel manufacture’s recommendation. Real-time PCR amplifications were performed using the Light Cycler 480 system (Roche). RT-qPCR data was analyzed by the 2^–(∆Ct)^ method^69^ using ACTB (Beta actin) as reference gene to yield relative fold expression levels of the seven different genes. Statistical analysis was done using univariate and multivariate logistic regression, modeling the probability of CD. These analyses were stratified according to the CT values of ACTB from 28 to 31. Relative fold expression values for MTCL1, SH3PXD2A-AS1, CLCF1, and CD180 genes were below detection limit and hence they were excluded from further analysis.

### In situ hybridization (ISH)

ISH for mRNAs PI3, ANXA1 and VDR were performed on 5 µm thick FFPE sections using the RNAscope^®^ 2.5 HD (Advanced Cell Diagnostics, CA)^70^ as described by Anderson et al.^71^ on automated Ventana Discovery Ultra instrument. Probes used for PI3, ANXA1 and VDR mRNA were RNAscope^®^ 2.5 VS Probe- Hs-PI3 (Target region:2–564, 12 zz pairs), RNAscope^®^ 2.5 VS Probe- Hs-ANXA1 (Target region:142–1095, 20 zz pairs), RNAscope^®^ 2.5 VS Probe-Hs-VDR (Target region:161–1191, 20 zz pairs), respectively. Probes to housekeeping gene PPIB mRNA (Target region: 139–989, 16 zz pairs) and negative control bacteria dapB mRNA (Target region: 414–862, 10 zz pairs) were used as positive and negative control, respectively. An alkaline phosphatase-based signal amplification system was applied and detected using the chromogenic substrate fast red. Positive mRNA expression was visible as red signal dots. Digital whole slides were obtained using a 20X objective in a bright-field slide scanner (Axio Scan.Z1; Zeiss). The probes for housekeeping gene PPIB and the bacterial dapB were applied in a subset of cases.

### Ethics approval and consent to participate

The study has been approved by the National Ethical Committee (j. nr. H-20032221) and the Danish Data Protection Agency (P-2020-737). The need for consent was waived by Danish National Ethical committee as tissue used for the project is formalin fixed paraffin embedded tissue previously collected and stored in the clinical biobank (tissue archive) at the department of Pathology at Herlev Hospital. The study was conducted in accordance with the ethical standards, according to the declaration of Helsinki, and according to national and international guidelines.

### Supplementary Information

Supplementary Information 1.

Supplementary Information 2.

Supplementary Information 3.

## Data Availability

Transcript profiling: The National Center for Biotechnology Information Gene Expression Omnibus database accession number for data from the discovery study reported in this paper is GSE222070. Data transparency statement: The raw data generated during and/or analysed during the current study are not publicly available, which is in accordance with the rules concerning processing of personal data set out in the EU General Data Protection Regulation (GDPR) and the Danish Data Protection Act. Nonetheless, may a researcher have an interest in our data they are welcome to contact us and collaborate. The data that support the findings of this study can be requested from The National Secretariat for Bio- and Genome Bank Denmark, RBGB.sekretariat.herlev-og-gentofte-hospital@regionh.dk, Herlev Hospital, Borgmester Ib Juuls Vej 73, 2730 Herlev, Denmark. Normalized data of the gene expression from the discovery study is uploaded to gene expression omnibus with the reference number: GSE222070.
