# Supplementary material for: Mucosal expression of PI3, ANXA1, and VDR discriminates Crohn’s disease from ulcerative colitis
Source: Sci Rep. 2023 Oct 27;13:18421. doi: 10.1038/s41598-023-45569-3 (PMC10611705; doi:10.1038/s41598-023-45569-3)
Supplement: Supplementary file 1 — Supplementary Information 1. [file 41598_2023_45569_MOESM1_ESM.pdf]

Supplementary file legends

Supplementary file 1. List of primers used for the custom Quantinova LNA RT-qPCR panel in the validation study.

Supplementary file 1 : The primers used in the custom panel RT-qPCR

Assay/targets	Assay catalog number
PPC	HS_PPC_2467741 QuantiNova LNA PCR Reference Assay (SBH1218550)
QIC	HS_QIC_2467742 QuantiNova LNA PCR Reference Assay (SBH1218551)
HGDC	HS_HGDC_2467744 QuantiNova LNA PCR Reference Assay (SBH1218553)
ACTB	HS_ACTB_2475367 QuantiNova LNA PCR Reference Assay (SBH1220543)
RPLP0	HS_RPLP0_2476240 QuantiNova LNA PCR Reference Assay (SBH1220553)
PI3	HS_PI3_1386086 QuantiNova LNA PCR Assay (SBH0142302)
ANXA1	HS_ANXA1_1342825 QuantiNova LNA PCR Assay (SBH0099041)
VDR	HS_VDR_1885746 QuantiNova LNA PCR Assay (SBH0641867)
MTCL1	HS_MTCL1_1263018 QuantiNova LNA PCR Assay (SBH0019234)
SH3PXD2A-AS1	HS_SH3PXD2A-AS1_1571027 QuantiNova LNA PCR Assay (SBH0327223)
CLCF1	HS_CLCF1_1669043 QuantiNova LNA PCR Assay (SBH0425229)
CD180	HS_CD180_2474682 QuantiNova LNA PCR Assay (SBH1219858)

Supplementary file 2. Results of testing 100 selected candidates from the discovery cohort in 3 data sets, GSE222070 (our own cohort), GSE117993, and GSE16879, using SAS. AUC - area under the receiver operating curve; CI - confidence interval. Given as a separate file (Supplementary file 2).

Supplementary file 3. Table of combined/individual AUC values at different ACTB Ct thresholds for three genes in discriminating CD from UC. P values for individual genes at different thresholds are shown in column 3.

Supplementary file 3 : Individual RT-qPCR AUCs and P values table

	ACTB Threshold	AUC for model	P value ANXA1/PI3/VDR
All 3 genes together. Age and sex included	No threshold	0.61	0.42/0.91/0.13
	<32	0.65	0.18/0.91/0.19
	<31	0.69	0.16/0.79/0.08
	<30	0.73	0.15/0.66/0.08
	<29	0.84	0.12/0.34/0.02
	<28	0.92	0.04/0.45/0.04
	<27	0.96	0.07/0.53/0.04
Univariate (ANXA1/PI3/VDR)	No threshold	0.45 /0.50 /0.59	0.99/0.69/0.18
	<32	0.60 /0.53 /0.58	0.27/0.88/0.26
	<31	0.61 /0.55 /0.63	0.27/0.77/0.12
	<30	0.66 /0.50 /0.67	0.23/0.85/0.07
	<29	0.67 /0.53 /0.77	0.27/0.59/0.01
	<28	0.74 /0.53 /0.80	0.14/0.96/0.01
	<27	0.84 /0.58 /0.81	0.04/0.46/0.02

Supplementary file 4. Complete list of gene ontology results for the functional analysis is presented in supplementary file 4. Given as a separate file (Supplementary file 4).
