# Supplementary material for: Mucosal expression of PI3, ANXA1, and VDR discriminates Crohn’s disease from ulcerative colitis
Source: Sci Rep. 2023 Oct 27;13:18421. doi: 10.1038/s41598-023-45569-3 (PMC10611705; doi:10.1038/s41598-023-45569-3)
Supplement: Supplementary file 2 — Supplementary Information 2. [file 41598_2023_45569_MOESM2_ESM.pdf]

Dataset	Gene	Probe set	Odds Ratio	Lower 95% CI	Upper 95% CI	P-value	AUC
1:GSE22207	ABCC1		0.12	0.02	0.69	0.0174	0.79
2:GSE11799	ABCC1		0.65	0.37	1.13	0.1267	0.59
3:GSE16879	ABCC1	202804_at	0.22	0.09	0.52	0.0006	0.69
3:GSE16879	ABCC1	202805_s_at	0.31	0.16	0.60	0.0006	0.76
1:GSE22207	ADAM33		0.31	0.12	0.77	0.0114	0.84
2:GSE11799	ADAM33		1.27	0.85	1.91	0.2448	0.56
3:GSE16879	ADAM33	232570_s_at	0.98	0.73	1.31	0.8853	0.51
3:GSE16879	ADAM33	233868_x_at	0.99	0.69	1.41	0.9381	0.45
1:GSE22207	ADAM9		0.10	0.02	0.57	0.0092	0.83
2:GSE11799	ADAM9		0.51	0.29	0.90	0.0214	0.63
3:GSE16879	ADAM9	1555326_a_at	0.33	0.18	0.57	0.0001	0.73
3:GSE16879	ADAM9	1570042_a_at	1.13	0.79	1.60	0.5107	0.50
3:GSE16879	ADAM9	202381_at	0.24	0.10	0.61	0.0026	0.69
1:GSE22207	ANXA1		0.12	0.03	0.52	0.0046	0.85
2:GSE11799	ANXA1		0.56	0.40	0.80	0.0012	0.68
3:GSE16879	ANXA1	201012_at	0.59	0.46	0.75	<.0001	0.76
1:GSE22207	ANXA3		0.35	0.15	0.82	0.0160	0.76
2:GSE11799	ANXA3		0.54	0.30	0.96	0.0351	0.62
3:GSE16879	ANXA3	209369_at	0.31	0.16	0.60	0.0004	0.70
1:GSE22207	ARRDC3		0.11	0.02	0.62	0.0122	0.83
2:GSE11799	ARRDC3		0.77	0.43	1.35	0.3603	0.58
3:GSE16879	ARRDC3	224797_at	0.21	0.10	0.41	<.0001	0.76
1:GSE22207	ATP8B2		0.16	0.04	0.63	0.0089	0.82
2:GSE11799	ATP8B2		0.70	0.44	1.10	0.1234	0.60
3:GSE16879	ATP8B2	216873_s_at	0.52	0.28	0.99	0.0475	0.60
3:GSE16879	ATP8B2	226771_at	0.74	0.55	0.99	0.0411	0.61
1:GSE22207	BHLHA15		0.25	0.09	0.66	0.0053	0.89
2:GSE11799	BHLHA15		0.57	0.39	0.83	0.0037	0.69
3:GSE16879	BHLHA15	235965_at	0.64	0.45	0.91	0.0130	0.67
1:GSE22207	BHLHE41		0.30	0.11	0.81	0.0177	0.82
2:GSE11799	BHLHE41		0.92	0.59	1.43	0.7022	0.52
3:GSE16879	BHLHE41	221530_s_at	0.35	0.20	0.59	<.0001	0.72
3:GSE16879	BHLHE41	223185_s_at	0.51	0.35	0.73	0.0003	0.71
3:GSE16879	BHLHE41	231242_at	0.96	0.72	1.27	0.7693	0.50
3:GSE16879	BHLHE41	231243_s_at	0.56	0.36	0.88	0.0111	0.65
1:GSE22207	BIN2		0.14	0.04	0.56	0.0050	0.80
2:GSE11799	BIN2		0.58	0.30	1.13	0.1107	0.59
3:GSE16879	BIN2	219191_s_at	0.76	0.58	0.98	0.0375	0.60
1:GSE22207	BTG2		0.19	0.05	0.67	0.0095	0.82
2:GSE11799	BTG2		0.74	0.43	1.27	0.2693	0.59

Dataset	Gene	Probe set	Odds Ratio	Lower 95% CI	Upper 95% CI	P-value	AUC
3:GSE16879	BTG2	201235_s_at	0.64	0.43	0.94	0.0224	0.62
3:GSE16879	BTG2	201236_s_at	0.40	0.22	0.72	0.0023	0.67
1:GSE22207	C21orf33		8.29	1.75	39.28	0.0077	0.83
2:GSE11799	C21orf33		0.71	0.52	0.98	0.0342	0.62
1:GSE22207	CBX4		0.18	0.05	0.67	0.0105	0.81
2:GSE11799	CBX4		0.45	0.20	1.02	0.0568	0.62
3:GSE16879	CBX4	206724_at	0.54	0.35	0.84	0.0065	0.66
3:GSE16879	CBX4	227558_at	0.36	0.18	0.71	0.0034	0.68
1:GSE22207	CD180		0.31	0.13	0.74	0.0085	0.86
2:GSE11799	CD180		0.37	0.23	0.60	<.0001	0.72
3:GSE16879	CD180	206206_at	0.75	0.59	0.95	0.0172	0.63
1:GSE22207	CLCF1		0.35	0.15	0.79	0.0120	0.83
2:GSE11799	CLCF1		0.60	0.36	0.99	0.0440	0.61
3:GSE16879	CLCF1	219500_at	0.23	0.12	0.47	<.0001	0.75
1:GSE22207	CNKSR3		3.94	1.31	11.79	0.0143	0.76
2:GSE11799	CNKSR3		2.76	1.46	5.20	0.0017	0.69
3:GSE16879	CNKSR3	1569331_at	1.01	0.81	1.27	0.9044	0.50
3:GSE16879	CNKSR3	227481_at	3.46	1.78	6.73	0.0003	0.73
3:GSE16879	CNKSR3	237302_at	1.25	0.96	1.63	0.0939	0.61
3:GSE16879	CNKSR3	242394_at	1.20	0.85	1.67	0.2997	0.58
1:GSE22207	COL3A1		0.27	0.09	0.78	0.0158	0.81
2:GSE11799	COL3A1		0.65	0.42	1.01	0.0577	0.63
3:GSE16879	COL3A1	201852_x_at	0.76	0.60	0.97	0.0246	0.63
3:GSE16879	COL3A1	211161_s_at	0.71	0.53	0.94	0.0161	0.64
3:GSE16879	COL3A1	215076_s_at	0.74	0.56	0.97	0.0269	0.62
1:GSE22207	CPEB4		0.04	0.00	0.46	0.0091	0.82
2:GSE11799	CPEB4		0.80	0.53	1.21	0.2966	0.58
3:GSE16879	CPEB4	224828_at	0.67	0.31	1.44	0.3067	0.55
3:GSE16879	CPEB4	224829_at	0.54	0.25	1.14	0.1060	0.57
3:GSE16879	CPEB4	224831_at	0.42	0.21	0.83	0.0123	0.63
1:GSE22207	CR1		0.35	0.15	0.83	0.0163	0.81
2:GSE11799	CR1		0.76	0.59	0.99	0.0433	0.61
3:GSE16879	CR1	206244_at	0.87	0.69	1.09	0.2302	0.59
3:GSE16879	CR1	208488_s_at	0.87	0.66	1.15	0.3249	0.55
3:GSE16879	CR1	217484_at	0.86	0.49	1.53	0.6107	0.54
3:GSE16879	CR1	217552_x_at	0.88	0.71	1.10	0.2621	0.59
3:GSE16879	CR1	239205_s_at	0.93	0.72	1.20	0.5768	0.53
3:GSE16879	CR1	244313_at	0.76	0.57	1.02	0.0703	0.59
1:GSE22207	CYTH4		0.28	0.10	0.74	0.0101	0.83
2:GSE11799	CYTH4		0.55	0.33	0.91	0.0212	0.63

Dataset	Gene	Probe set	Odds Ratio	Lower 95% CI	Upper 95% CI	P-value	AUC
3:GSE16879	CYTH4	1554335_at	0.87	0.68	1.12	0.2743	0.57
3:GSE16879	CYTH4	211521_s_at	0.99	0.65	1.51	0.9699	0.47
3:GSE16879	CYTH4	219183_s_at	0.81	0.51	1.31	0.3981	0.55
1:GSE22207	DECR2		4.27	1.30	14.09	0.0170	0.80
2:GSE11799	DECR2		3.65	1.62	8.26	0.0018	0.69
3:GSE16879	DECR2	219664_s_at	11.86	4.28	32.82	<.0001	0.81
1:GSE22207	DNM1		0.31	0.12	0.80	0.0160	0.81
2:GSE11799	DNM1		0.83	0.52	1.31	0.4283	0.54
3:GSE16879	DNM1	215116_s_at	0.87	0.67	1.14	0.3264	0.55
1:GSE22207	DTX3		0.25	0.08	0.75	0.0130	0.76
2:GSE11799	DTX3		0.98	0.51	1.88	0.9529	0.50
3:GSE16879	DTX3	221835_at	0.95	0.67	1.33	0.7552	0.52
3:GSE16879	DTX3	235721_at	0.64	0.45	0.90	0.0109	0.64
3:GSE16879	DTX3	49049_at	0.73	0.52	1.03	0.0699	0.60
3:GSE16879	DTX3	49051_g_at	1.08	0.83	1.41	0.5830	0.53
1:GSE22207	EDEM1		0.12	0.02	0.59	0.0099	0.81
2:GSE11799	EDEM1		0.62	0.33	1.16	0.1369	0.60
3:GSE16879	EDEM1	203279_at	0.11	0.03	0.36	0.0003	0.74
1:GSE22207	ELL2		0.14	0.03	0.71	0.0173	0.77
2:GSE11799	ELL2		0.53	0.33	0.86	0.0104	0.64
3:GSE16879	ELL2	214446_at	0.53	0.35	0.81	0.0033	0.72
3:GSE16879	ELL2	226099_at	0.41	0.23	0.71	0.0014	0.67
3:GSE16879	ELL2	226982_at	0.52	0.26	1.03	0.0605	0.60
3:GSE16879	ELL2	240038_at	0.59	0.33	1.04	0.0677	0.60
1:GSE22207	EMD		0.31	0.12	0.80	0.0161	0.79
2:GSE11799	EMD		0.27	0.05	1.54	0.1393	0.57
3:GSE16879	EMD	209477_at	0.43	0.19	0.99	0.0469	0.60
2:GSE11799	ERO1A		0.45	0.30	0.66	<.0001	0.74
3:GSE16879	ERO1A	218498_s_at	0.40	0.27	0.59	<.0001	0.75
3:GSE16879	ERO1A	222646_s_at	0.49	0.35	0.69	<.0001	0.73
3:GSE16879	ERO1A	225750_at	0.24	0.13	0.42	<.0001	0.77
1:GSE22207	ERO1L		0.18	0.05	0.67	0.0104	0.81
1:GSE22207	FAM100B		12.17	1.64	90.14	0.0144	0.81
1:GSE22207	FAM227B		0.34	0.15	0.77	0.0094	0.86
2:GSE11799	FAM227B		1.04	0.52	2.10	0.9079	0.53
3:GSE16879	FAM227B	1553799_at	1.10	0.89	1.37	0.3820	0.54
3:GSE16879	FAM227B	1557429_s_at	1.19	0.92	1.55	0.1926	0.56
1:GSE22207	FAM46C		0.04	0.00	0.41	0.0069	0.89
2:GSE11799	FAM46C		0.37	0.19	0.73	0.0044	0.66
1:GSE22207	FCRL5		0.20	0.06	0.67	0.0095	0.87

Dataset	Gene	Probe set	Odds Ratio	Lower 95% CI	Upper 95% CI	P-value	AUC
2:GSE11799	FCRL5		0.46	0.31	0.68	0.0001	0.71
3:GSE16879	FCRL5	1555799_at	0.85	0.67	1.09	0.1930	0.57
3:GSE16879	FCRL5	224404_s_at	0.79	0.65	0.97	0.0272	0.66
3:GSE16879	FCRL5	224405_at	0.74	0.59	0.95	0.0163	0.69
3:GSE16879	FCRL5	224406_s_at	0.73	0.59	0.92	0.0079	0.66
3:GSE16879	FCRL5	231647_s_at	0.71	0.57	0.88	0.0018	0.69
1:GSE22207	FGF7		0.31	0.13	0.73	0.0073	0.81
2:GSE11799	FGF7		0.92	0.69	1.23	0.5792	0.55
3:GSE16879	FGF7	1554741_s_at	0.77	0.57	1.03	0.0808	0.62
3:GSE16879	FGF7	1555102_at	1.04	0.84	1.28	0.7450	0.51
3:GSE16879	FGF7	1555103_s_at	0.97	0.78	1.20	0.7674	0.49
3:GSE16879	FGF7	205782_at	0.98	0.68	1.39	0.8910	0.52
2:GSE11799	GCN1		1.55	0.76	3.16	0.2232	0.59
3:GSE16879	GCN1	212139_at	0.12	0.03	0.53	0.0050	0.67
3:GSE16879	GCN1	216232_s_at	0.07	0.01	0.37	0.0018	0.66
1:GSE22207	GCN1L1		0.13	0.02	0.69	0.0173	0.78
1:GSE22207	GNA15		0.33	0.13	0.83	0.0184	0.79
2:GSE11799	GNA15		0.38	0.23	0.62	0.0001	0.74
3:GSE16879	GNA15	205349_at	0.49	0.33	0.73	0.0004	0.72
1:GSE22207	GPR63		0.32	0.13	0.80	0.0150	0.80
2:GSE11799	GPR63		1.39	0.89	2.17	0.1461	0.58
3:GSE16879	GPR63	220993_s_at	0.98	0.72	1.32	0.8823	0.51
1:GSE22207	GPRC5A		0.34	0.15	0.79	0.0112	0.85
2:GSE11799	GPRC5A		0.82	0.53	1.25	0.3508	0.56
3:GSE16879	GPRC5A	203108_at	0.25	0.14	0.46	<.0001	0.77
3:GSE16879	GPRC5A	212444_at	0.15	0.07	0.32	<.0001	0.82
3:GSE16879	GPRC5A	235563_at	0.52	0.38	0.73	0.0001	0.74
1:GSE22207	HERPUD1		0.06	0.01	0.49	0.0087	0.82
2:GSE11799	HERPUD1		0.37	0.16	0.85	0.0192	0.64
3:GSE16879	HERPUD1	1569380_a_at	0.96	0.73	1.27	0.7740	0.51
3:GSE16879	HERPUD1	217168_s_at	0.23	0.09	0.59	0.0021	0.68
1:GSE22207	HGF		0.20	0.06	0.66	0.0088	0.89
2:GSE11799	HGF		0.75	0.55	1.03	0.0745	0.60
3:GSE16879	HGF	209960_at	0.74	0.57	0.96	0.0243	0.63
3:GSE16879	HGF	209961_s_at	0.78	0.55	1.11	0.1738	0.62
3:GSE16879	HGF	210755_at	0.89	0.67	1.17	0.3900	0.56
3:GSE16879	HGF	210997_at	0.86	0.72	1.04	0.1152	0.60
3:GSE16879	HGF	210998_s_at	0.92	0.73	1.15	0.4394	0.55
1:GSE22207	HMGB2		6.78	1.42	32.25	0.0162	0.79
2:GSE11799	HMGB2		1.20	0.53	2.73	0.6616	0.52

Dataset	Gene	Probe set	Odds Ratio	Lower 95% CI	Upper 95% CI	P-value	AUC
3:GSE16879	HMGB2	208808_s_at	0.36	0.16	0.85	0.0187	0.64
1:GSE22207	IGLL3P		0.19	0.06	0.59	0.0042	0.85
2:GSE11799	IGLL3P		0.68	0.54	0.87	0.0020	0.73
3:GSE16879	IGLL3P	215946_x_at	0.69	0.55	0.87	0.0017	0.67
1:GSE22207	IL6R		0.16	0.04	0.65	0.0103	0.82
2:GSE11799	IL6R		2.30	1.20	4.40	0.0119	0.62
3:GSE16879	IL6R	205945_at	3.46	1.51	7.93	0.0034	0.65
3:GSE16879	IL6R	217489_s_at	1.20	0.86	1.68	0.2855	0.56
3:GSE16879	IL6R	226333_at	3.74	1.50	9.29	0.0046	0.68
1:GSE22207	IRF4		0.26	0.10	0.68	0.0061	0.85
2:GSE11799	IRF4		0.51	0.33	0.79	0.0024	0.67
3:GSE16879	IRF4	204562_at	0.57	0.40	0.81	0.0019	0.69
3:GSE16879	IRF4	216986_s_at	0.97	0.71	1.31	0.8246	0.51
3:GSE16879	IRF4	216987_at	0.80	0.61	1.06	0.1195	0.59
1:GSE22207	ITGA6		0.10	0.02	0.65	0.0156	0.81
2:GSE11799	ITGA6		0.70	0.38	1.27	0.2404	0.57
3:GSE16879	ITGA6	201656_at	0.49	0.19	1.29	0.1485	0.57
3:GSE16879	ITGA6	215177_s_at	0.33	0.18	0.61	0.0005	0.69
1:GSE22207	ITPKB		0.27	0.10	0.75	0.0115	0.78
2:GSE11799	ITPKB		0.81	0.44	1.49	0.4908	0.52
3:GSE16879	ITPKB	1554306_at	1.11	0.84	1.46	0.4643	0.54
3:GSE16879	ITPKB	203723_at	1.01	0.69	1.49	0.9540	0.50
1:GSE22207	KIAA0040		0.09	0.01	0.62	0.0139	0.82
2:GSE11799	KIAA0040		0.73	0.40	1.30	0.2848	0.58
3:GSE16879	KIAA0040	203143_s_at	0.18	0.07	0.42	<.0001	0.74
3:GSE16879	KIAA0040	203144_s_at	0.44	0.25	0.78	0.0046	0.69
1:GSE22207	KLF2		0.24	0.08	0.72	0.0115	0.81
2:GSE11799	KLF2		0.78	0.50	1.24	0.2959	0.55
3:GSE16879	KLF2	219371_s_at	0.28	0.16	0.48	<.0001	0.77
3:GSE16879	KLF2	226646_at	0.84	0.59	1.18	0.3049	0.55
1:GSE22207	KLHL5		0.23	0.07	0.78	0.0185	0.81
2:GSE11799	KLHL5		0.59	0.39	0.91	0.0164	0.64
3:GSE16879	KLHL5	226001_at	0.88	0.66	1.18	0.3957	0.54
3:GSE16879	KLHL5	227565_at	1.12	0.72	1.73	0.6168	0.53
3:GSE16879	KLHL5	232297_at	1.00	0.68	1.47	0.9971	0.51
3:GSE16879	KLHL5	233866_at	0.83	0.64	1.09	0.1800	0.58
1:GSE22207	LAMC1		0.18	0.04	0.75	0.0181	0.82
2:GSE11799	LAMC1		0.83	0.53	1.29	0.4088	0.57
3:GSE16879	LAMC1	200770_s_at	0.48	0.30	0.77	0.0021	0.69
3:GSE16879	LAMC1	200771_at	0.58	0.36	0.94	0.0261	0.66

Dataset	Gene	Probe set	Odds Ratio	Lower 95% CI	Upper 95% CI	P-value	AUC
1:GSE22207	LEPR		0.31	0.12	0.80	0.0150	0.88
2:GSE11799	LEPR		1.07	0.67	1.71	0.7801	0.52
3:GSE16879	LEPR	1556997_a_at	0.95	0.75	1.19	0.6419	0.51
3:GSE16879	LEPR	207255_at	0.89	0.64	1.25	0.5142	0.49
3:GSE16879	LEPR	209894_at	0.79	0.47	1.34	0.3859	0.56
3:GSE16879	LEPR	211354_s_at	0.83	0.61	1.11	0.2106	0.56
3:GSE16879	LEPR	211355_x_at	0.89	0.59	1.33	0.5582	0.53
3:GSE16879	LEPR	211356_x_at	0.76	0.45	1.28	0.2975	0.53
1:GSE22207	LINC00167		3.09	1.33	7.17	0.0088	0.79
2:GSE11799	LINC00167		1.12	0.90	1.38	0.3068	0.57
3:GSE16879	LINC00167	1562413_at	0.85	0.65	1.11	0.2257	0.57
1:GSE22207	LOC392196		2.78	1.31	5.92	0.0080	0.82
1:GSE22207	ME1		0.15	0.04	0.64	0.0105	0.86
2:GSE11799	ME1		0.49	0.30	0.79	0.0038	0.66
3:GSE16879	ME1	204058_at	0.43	0.27	0.68	0.0003	0.73
3:GSE16879	ME1	204059_s_at	0.38	0.22	0.64	0.0003	0.73
1:GSE22207	MRVI1		0.17	0.05	0.67	0.0111	0.82
2:GSE11799	MRVI1		0.87	0.51	1.47	0.5989	0.53
1:GSE22207	MT1E		3.05	1.27	7.34	0.0128	0.79
2:GSE11799	MT1E		1.69	1.16	2.48	0.0064	0.65
3:GSE16879	MT1E	212859_x_at	1.28	0.86	1.91	0.2310	0.59
2:GSE11799	MTCL1		0.60	0.39	0.91	0.0167	0.63
3:GSE16879	MTCL1	213358_at	0.33	0.20	0.54	<.0001	0.76
1:GSE22207	MTMR10		21.91	2.41	199.11	0.0061	0.88
2:GSE11799	MTMR10		2.61	1.18	5.80	0.0184	0.62
3:GSE16879	MTMR10	220286_at	0.79	0.55	1.13	0.1915	0.57
3:GSE16879	MTMR10	225810_at	2.12	0.57	7.80	0.2602	0.57
1:GSE22207	MYEOV		0.36	0.16	0.79	0.0111	0.82
2:GSE11799	MYEOV		0.61	0.44	0.84	0.0025	0.67
3:GSE16879	MYEOV	227342_s_at	0.36	0.19	0.68	0.0015	0.67
1:GSE22207	MZB1		0.28	0.11	0.73	0.0090	0.80
2:GSE11799	MZB1		0.38	0.25	0.59	<.0001	0.74
3:GSE16879	MZB1	221286_s_at	0.69	0.56	0.85	0.0006	0.71
3:GSE16879	MZB1	223565_at	0.73	0.59	0.89	0.0026	0.68
1:GSE22207	NLRX1		0.13	0.03	0.67	0.0149	0.81
2:GSE11799	NLRX1		1.76	0.63	4.91	0.2773	0.57
3:GSE16879	NLRX1	1553695_a_at	0.72	0.53	0.97	0.0328	0.64
3:GSE16879	NLRX1	219680_at	0.61	0.30	1.23	0.1661	0.58
1:GSE22207	PDK1		0.24	0.08	0.73	0.0122	0.87
2:GSE11799	PDK1		0.40	0.23	0.70	0.0015	0.68

Dataset	Gene	Probe set	Odds Ratio	Lower 95% CI	Upper 95% CI	P-value	AUC
3:GSE16879	PDK1	206686_at	0.46	0.31	0.67	<.0001	0.75
3:GSE16879	PDK1	226452_at	0.19	0.09	0.38	<.0001	0.79
3:GSE16879	PDK1	239798_at	0.63	0.41	0.97	0.0340	0.61
1:GSE22207	PHF21A		0.27	0.09	0.79	0.0172	0.77
2:GSE11799	PHF21A		0.74	0.42	1.33	0.3162	0.54
3:GSE16879	PHF21A	1554153_a_at	0.77	0.47	1.26	0.2957	0.53
3:GSE16879	PHF21A	203278_s_at	0.49	0.25	0.96	0.0374	0.60
3:GSE16879	PHF21A	227090_at	1.22	0.71	2.09	0.4770	0.49
1:GSE22207	PI3		0.21	0.07	0.61	0.0039	0.90
2:GSE11799	PI3		0.42	0.30	0.59	<.0001	0.83
3:GSE16879	PI3	203691_at	0.56	0.44	0.72	<.0001	0.79
3:GSE16879	PI3	41469_at	0.52	0.39	0.69	<.0001	0.79
1:GSE22207	PITPNC1		0.05	0.01	0.37	0.0041	0.88
2:GSE11799	PITPNC1		0.16	0.07	0.35	<.0001	0.78
3:GSE16879	PITPNC1	1568949_at	0.51	0.33	0.80	0.0032	0.70
3:GSE16879	PITPNC1	219155_at	0.25	0.14	0.44	<.0001	0.79
3:GSE16879	PITPNC1	229414_at	0.43	0.26	0.70	0.0007	0.71
3:GSE16879	PITPNC1	238649_at	0.28	0.15	0.53	<.0001	0.74
1:GSE22207	PLEKHG2		0.30	0.11	0.82	0.0185	0.77
2:GSE11799	PLEKHG2		0.62	0.39	0.98	0.0407	0.62
3:GSE16879	PLEKHG2	1558407_at	1.67	0.99	2.82	0.0558	0.61
3:GSE16879	PLEKHG2	225979_at	0.85	0.67	1.07	0.1639	0.57
3:GSE16879	PLEKHG2	233986_s_at	0.84	0.68	1.04	0.1067	0.59
1:GSE22207	PPARD		0.10	0.02	0.59	0.0111	0.82
2:GSE11799	PPARD		1.25	0.63	2.46	0.5223	0.55
3:GSE16879	PPARD	208044_s_at	0.43	0.22	0.84	0.0142	0.63
3:GSE16879	PPARD	210636_at	0.95	0.74	1.23	0.7001	0.52
3:GSE16879	PPARD	37152_at	0.22	0.10	0.48	0.0001	0.73
1:GSE22207	PRDM15		0.26	0.09	0.75	0.0133	0.86
2:GSE11799	PRDM15		0.60	0.36	1.01	0.0547	0.61
3:GSE16879	PRDM15	230777_s_at	0.58	0.28	1.18	0.1298	0.62
3:GSE16879	PRDM15	231931_at	0.81	0.60	1.10	0.1754	0.59
3:GSE16879	PRDM15	234524_at	0.98	0.67	1.43	0.9186	0.55
3:GSE16879	PRDM15	236061_at	1.09	0.80	1.47	0.5918	0.55
1:GSE22207	PRSS22		0.37	0.17	0.77	0.0076	0.82
2:GSE11799	PRSS22		0.63	0.42	0.96	0.0293	0.63
3:GSE16879	PRSS22	205847_at	0.29	0.15	0.56	0.0002	0.70
1:GSE22207	RBM12		0.18	0.05	0.69	0.0126	0.81
2:GSE11799	RBM12		1.19	0.68	2.08	0.5413	0.54
3:GSE16879	RBM12	212168_at	0.02	0.00	0.17	0.0003	0.70

Dataset	Gene	Probe set	Odds Ratio	Lower 95% CI	Upper 95% CI	P-value	AUC
3:GSE16879	RBM12	212170_at	0.33	0.14	0.79	0.0124	0.65
1:GSE22207	RBPMS		0.10	0.02	0.50	0.0048	0.85
2:GSE11799	RBPMS		0.20	0.10	0.41	<.0001	0.76
3:GSE16879	RBPMS	1557223_at	0.80	0.53	1.20	0.2871	0.55
3:GSE16879	RBPMS	207836_s_at	0.46	0.32	0.67	<.0001	0.76
3:GSE16879	RBPMS	207837_at	0.81	0.64	1.03	0.0846	0.59
3:GSE16879	RBPMS	209487_at	0.39	0.25	0.59	<.0001	0.76
3:GSE16879	RBPMS	209488_s_at	0.42	0.29	0.63	<.0001	0.76
3:GSE16879	RBPMS	231961_at	0.92	0.69	1.24	0.5915	0.56
1:GSE22207	RDX		0.14	0.03	0.66	0.0123	0.82
2:GSE11799	RDX		1.17	0.69	1.98	0.5707	0.51
3:GSE16879	RDX	204969_s_at	0.59	0.38	0.92	0.0186	0.64
3:GSE16879	RDX	212397_at	0.62	0.43	0.89	0.0100	0.65
3:GSE16879	RDX	212398_at	0.59	0.36	0.96	0.0331	0.61
3:GSE16879	RDX	244739_at	0.90	0.68	1.20	0.4821	0.54
1:GSE22207	RHOJ		0.16	0.04	0.72	0.0172	0.85
2:GSE11799	RHOJ		0.65	0.37	1.14	0.1325	0.60
3:GSE16879	RHOJ	1555233_at	0.99	0.80	1.23	0.9433	0.51
3:GSE16879	RHOJ	1555234_a_at	0.86	0.65	1.15	0.3135	0.55
3:GSE16879	RHOJ	235131_at	1.20	0.64	2.24	0.5758	0.55
3:GSE16879	RHOJ	235489_at	0.76	0.55	1.06	0.1034	0.64
3:GSE16879	RHOJ	238905_at	0.61	0.37	1.00	0.0487	0.60
3:GSE16879	RHOJ	238906_s_at	0.91	0.70	1.19	0.5053	0.55
3:GSE16879	RHOJ	243481_at	0.94	0.68	1.30	0.6911	0.51
1:GSE22207	RPN2		0.11	0.02	0.62	0.0123	0.78
2:GSE11799	RPN2		0.21	0.06	0.67	0.0082	0.65
3:GSE16879	RPN2	208689_s_at	0.12	0.03	0.42	0.0011	0.69
3:GSE16879	RPN2	213399_x_at	0.28	0.08	0.91	0.0340	0.63
3:GSE16879	RPN2	213491_x_at	0.27	0.09	0.82	0.0205	0.65
1:GSE22207	SDSL		3.62	1.34	9.79	0.0111	0.81
2:GSE11799	SDSL		4.17	1.65	10.55	0.0025	0.69
3:GSE16879	SDSL	228274_at	1.95	1.26	3.01	0.0027	0.69
1:GSE22207	SEC14L1		0.11	0.02	0.69	0.0185	0.79
2:GSE11799	SEC14L1		0.51	0.26	0.98	0.0441	0.61
3:GSE16879	SEC14L1	202082_s_at	0.44	0.24	0.80	0.0071	0.65
3:GSE16879	SEC14L1	202083_s_at	0.47	0.29	0.77	0.0029	0.67
3:GSE16879	SEC14L1	202084_s_at	0.27	0.12	0.58	0.0008	0.69
1:GSE22207	SEL1L3		0.06	0.01	0.55	0.0129	0.79
2:GSE11799	SEL1L3		0.10	0.03	0.39	0.0009	0.68
3:GSE16879	SEL1L3	212311_at	0.18	0.05	0.61	0.0061	0.65

Dataset	Gene	Probe set	Odds Ratio	Lower 95% CI	Upper 95% CI	P-value	AUC
3:GSE16879	SEL1L3	212314_at	1.27	0.33	4.97	0.7295	0.51
3:GSE16879	SEL1L3	235353_at	0.58	0.29	1.18	0.1350	0.58
1:GSE22207	SH3PXD2A		0.29	0.13	0.65	0.0029	0.93
2:GSE11799	SH3PXD2A		1.96	1.06	3.63	0.0316	0.63
3:GSE16879	SH3PXD2A	207661_s_at	0.99	0.68	1.44	0.9515	0.50
3:GSE16879	SH3PXD2A	213252_at	0.95	0.52	1.73	0.8596	0.52
3:GSE16879	SH3PXD2A	224817_at	0.19	0.06	0.62	0.0059	0.63
1:GSE22207	SMARCD1		0.07	0.01	0.59	0.0147	0.81
2:GSE11799	SMARCD1		1.03	0.39	2.73	0.9504	0.52
3:GSE16879	SMARCD1	203183_s_at	0.69	0.35	1.33	0.2653	0.56
3:GSE16879	SMARCD1	209518_at	0.76	0.36	1.59	0.4683	0.52
1:GSE22207	SNPH		0.24	0.07	0.77	0.0170	0.83
2:GSE11799	SNPH		0.45	0.30	0.68	0.0001	0.72
3:GSE16879	SNPH	205104_at	0.43	0.28	0.65	<.0001	0.76
1:GSE22207	SNRNP48		7.23	1.42	36.87	0.0174	0.75
2:GSE11799	SNRNP48		1.78	0.89	3.52	0.1007	0.62
3:GSE16879	SNRNP48	1553274_a_at	2.19	0.78	6.13	0.1363	0.58
3:GSE16879	SNRNP48	226263_at	4.64	1.75	12.35	0.0021	0.70
1:GSE22207	SOGA2		0.08	0.01	0.58	0.0121	0.90
1:GSE22207	SPAG4		0.19	0.05	0.66	0.0090	0.88
2:GSE11799	SPAG4		0.50	0.33	0.76	0.0012	0.69
3:GSE16879	SPAG4	219888_at	0.74	0.57	0.97	0.0274	0.65
1:GSE22207	SPNS2		0.26	0.09	0.76	0.0137	0.77
2:GSE11799	SPNS2		0.42	0.26	0.69	0.0005	0.70
3:GSE16879	SPNS2	225671_at	0.73	0.44	1.21	0.2248	0.55
1:GSE22207	SSH1		0.20	0.05	0.75	0.0169	0.83
2:GSE11799	SSH1		1.16	0.74	1.83	0.5173	0.52
3:GSE16879	SSH1	1554274_a_at	0.96	0.66	1.39	0.8096	0.51
3:GSE16879	SSH1	1555624_a_at	1.16	0.85	1.59	0.3431	0.55
3:GSE16879	SSH1	221752_at	0.32	0.14	0.73	0.0064	0.65
3:GSE16879	SSH1	221753_at	0.46	0.17	1.24	0.1259	0.59
1:GSE22207	TACC1		0.11	0.02	0.70	0.0186	0.75
2:GSE11799	TACC1		1.40	0.85	2.31	0.1913	0.57
3:GSE16879	TACC1	1554690_a_at	0.47	0.26	0.86	0.0133	0.63
3:GSE16879	TACC1	200911_s_at	0.22	0.08	0.56	0.0015	0.68
3:GSE16879	TACC1	217437_s_at	0.34	0.16	0.69	0.0032	0.67
3:GSE16879	TACC1	242290_at	0.94	0.59	1.49	0.7793	0.52
1:GSE22207	TCF4		0.17	0.04	0.64	0.0090	0.85
2:GSE11799	TCF4		0.72	0.46	1.14	0.1571	0.59
3:GSE16879	TCF4	203753_at	0.64	0.43	0.96	0.0293	0.62

Dataset	Gene	Probe set	Odds Ratio	Lower 95% CI	Upper 95% CI	P-value	AUC
3:GSE16879	TCF4	212382_at	0.72	0.50	1.05	0.0864	0.59
3:GSE16879	TCF4	212385_at	0.72	0.53	0.96	0.0269	0.64
3:GSE16879	TCF4	212386_at	0.56	0.36	0.87	0.0091	0.66
3:GSE16879	TCF4	212387_at	0.55	0.37	0.83	0.0047	0.66
3:GSE16879	TCF4	213891_s_at	0.64	0.45	0.92	0.0172	0.65
3:GSE16879	TCF4	222146_s_at	0.57	0.37	0.86	0.0075	0.68
3:GSE16879	TCF4	228837_at	0.70	0.51	0.96	0.0251	0.65
1:GSE22207	THBD		0.15	0.03	0.61	0.0086	0.81
2:GSE11799	THBD		0.59	0.40	0.88	0.0092	0.64
3:GSE16879	THBD	203887_s_at	0.65	0.47	0.89	0.0079	0.67
3:GSE16879	THBD	203888_at	0.69	0.51	0.92	0.0122	0.65
3:GSE16879	THBD	237252_at	0.80	0.63	1.02	0.0686	0.60
1:GSE22207	TLR1		0.25	0.08	0.74	0.0121	0.83
2:GSE11799	TLR1		0.84	0.59	1.19	0.3252	0.56
3:GSE16879	TLR1	210176_at	0.93	0.66	1.32	0.6959	0.54
1:GSE22207	TMEM156		0.31	0.13	0.78	0.0125	0.79
2:GSE11799	TMEM156		0.55	0.34	0.90	0.0165	0.63
3:GSE16879	TMEM156	220169_at	0.75	0.54	1.02	0.0688	0.59
3:GSE16879	TMEM156	241844_x_at	0.71	0.52	0.96	0.0243	0.65
1:GSE22207	TMEM159		0.23	0.07	0.75	0.0145	0.76
2:GSE11799	TMEM159		0.40	0.14	1.15	0.0885	0.57
3:GSE16879	TMEM159	213272_s_at	0.16	0.07	0.39	<.0001	0.74
1:GSE22207	TMEM184B		0.04	0.00	0.37	0.0046	0.88
2:GSE11799	TMEM184B		0.31	0.12	0.80	0.0157	0.63
3:GSE16879	TMEM184B	202027_at	0.41	0.13	1.25	0.1163	0.59
1:GSE22207	TRIOBP		0.07	0.01	0.61	0.0159	0.78
2:GSE11799	TRIOBP		1.22	0.63	2.37	0.5639	0.54
3:GSE16879	TRIOBP	1552332_at	0.52	0.33	0.81	0.0040	0.67
3:GSE16879	TRIOBP	1552334_at	0.23	0.09	0.59	0.0021	0.68
3:GSE16879	TRIOBP	202795_x_at	0.12	0.04	0.37	0.0002	0.70
3:GSE16879	TRIOBP	210276_s_at	0.06	0.02	0.23	<.0001	0.76
3:GSE16879	TRIOBP	216210_x_at	0.14	0.05	0.40	0.0003	0.70
3:GSE16879	TRIOBP	243690_at	0.49	0.25	0.98	0.0427	0.61
1:GSE22207	TUT1		6.89	1.58	30.09	0.0103	0.81
2:GSE11799	TUT1		2.68	0.91	7.90	0.0732	0.58
3:GSE16879	TUT1	218965_s_at	1.31	0.93	1.86	0.1190	0.54
1:GSE22207	TYW3		0.16	0.03	0.70	0.0156	0.83
2:GSE11799	TYW3		0.74	0.24	2.27	0.5935	0.53
3:GSE16879	TYW3	227141_at	0.08	0.02	0.27	<.0001	0.73
2:GSE11799	UBALD2		4.03	1.32	12.25	0.0142	0.65

Dataset	Gene	Probe set	Odds Ratio	Lower 95% CI	Upper 95% CI	P-value	AUC
3:GSE16879	UBALD2	224783_at	4.77	1.96	11.60	0.0006	0.70
3:GSE16879	UBALD2	224785_at	1.26	0.69	2.30	0.4544	0.53
1:GSE22207	UBE2J1		0.00	0.00	0.31	0.0123	0.90
2:GSE11799	UBE2J1		0.31	0.12	0.77	0.0119	0.66
3:GSE16879	UBE2J1	217823_s_at	0.27	0.10	0.74	0.0115	0.64
3:GSE16879	UBE2J1	217824_at	0.57	0.22	1.51	0.2619	0.56
3:GSE16879	UBE2J1	217825_s_at	0.18	0.05	0.65	0.0095	0.63
3:GSE16879	UBE2J1	217826_s_at	0.22	0.07	0.70	0.0105	0.66
3:GSE16879	UBE2J1	222435_s_at	0.14	0.05	0.40	0.0003	0.70
1:GSE22207	USO1		0.07	0.01	0.53	0.0099	0.85
2:GSE11799	USO1		1.14	0.65	2.00	0.6494	0.52
3:GSE16879	USO1	201831_s_at	0.31	0.08	1.22	0.0928	0.60
3:GSE16879	USO1	201832_s_at	0.22	0.06	0.75	0.0153	0.63
2:GSE11799	USP17L9P		1.00	.	.	.	0.51
1:GSE22207	USP36		11.45	1.75	75.15	0.0111	0.82
2:GSE11799	USP36		2.25	0.85	5.94	0.1005	0.58
3:GSE16879	USP36	1554836_at	0.79	0.61	1.02	0.0740	0.57
3:GSE16879	USP36	220370_s_at	0.63	0.35	1.13	0.1217	0.57
3:GSE16879	USP36	224978_s_at	0.21	0.09	0.50	0.0004	0.71
3:GSE16879	USP36	224979_s_at	0.56	0.38	0.84	0.0054	0.63
3:GSE16879	USP36	227093_at	0.37	0.12	1.14	0.0838	0.59
1:GSE22207	VDR		7.87	1.70	36.54	0.0084	0.81
2:GSE11799	VDR		4.76	2.33	9.71	<.0001	0.75
3:GSE16879	VDR	204253_s_at	2.66	1.19	5.93	0.0170	0.63
3:GSE16879	VDR	204254_s_at	7.42	3.13	17.61	<.0001	0.76
3:GSE16879	VDR	204255_s_at	3.56	1.62	7.85	0.0016	0.70
1:GSE22207	VIM		0.20	0.06	0.64	0.0065	0.85
2:GSE11799	VIM		0.49	0.25	0.99	0.0466	0.64
3:GSE16879	VIM	201426_s_at	0.73	0.54	0.99	0.0426	0.61
1:GSE22207	VPS4B		5.83	1.43	23.73	0.0138	0.80
2:GSE11799	VPS4B		2.81	1.30	6.08	0.0088	0.65
3:GSE16879	VPS4B	218171_at	2.15	0.64	7.23	0.2158	0.59
1:GSE22207	XBP1		0.17	0.05	0.62	0.0067	0.83
2:GSE11799	XBP1		0.21	0.10	0.43	<.0001	0.75
1:GSE22207	ZNF460		0.04	0.00	0.49	0.0127	0.81
2:GSE11799	ZNF460		0.92	0.63	1.34	0.6667	0.50
3:GSE16879	ZNF460	216279_at	0.65	0.42	1.02	0.0627	0.59
3:GSE16879	ZNF460	233175_at	0.92	0.67	1.26	0.5977	0.54
