# Supplementary material for: Mucosal expression of PI3, ANXA1, and VDR discriminates Crohn’s disease from ulcerative colitis
Source: Sci Rep. 2023 Oct 27;13:18421. doi: 10.1038/s41598-023-45569-3 (PMC10611705; doi:10.1038/s41598-023-45569-3)
Supplement: Supplementary file 3 — Supplementary Information 3. [file 41598_2023_45569_MOESM3_ESM.pdf]

Category	ID	Name	p-value	q-value Bonferroni	q-value FDR B&H	q-value FDR B&Y	Hit Count in Query List	Hit Count in Genome	Hit in Query List
GO: Molecular Function	GO:0038186	lithocholic acid receptor activity	4.02E-04	4.34E-02	1.63E-02	8.56E-02	1	1	VDR
GO: Molecular Function	GO:1902098	calcitriol binding	4.02E-04	4.34E-02	1.63E-02	8.56E-02	1	1	VDR
GO: Molecular Function	GO:0036121	double-stranded DNA helicase activity	1.21E-03	1.30E-01	1.63E-02	8.56E-02	1	3	ANXA1
GO: Molecular Function	GO:1902271	D3 vitamins binding	1.21E-03	1.30E-01	1.63E-02	8.56E-02	1	3	VDR
GO: Molecular Function	GO:0004471	malate dehydrogenase (decarboxylating) (NAD+) activity	1.21E-03	1.30E-01	1.63E-02	8.56E-02	1	3	ME1
GO: Molecular Function	GO:0004473	malate dehydrogenase (decarboxylating) (NADP+) activity	1.21E-03	1.30E-01	1.63E-02	8.56E-02	1	3	ME1
GO: Molecular Function	GO:0038181	bile acid receptor activity	1.21E-03	1.30E-01	1.63E-02	8.56E-02	1	3	VDR
GO: Molecular Function	GO:0070644	vitamin D response element binding	1.21E-03	1.30E-01	1.63E-02	8.56E-02	1	3	VDR
GO: Molecular Function	GO:0004470	malic enzyme activity	1.61E-03	1.74E-01	1.74E-02	9.13E-02	1	4	ME1
GO: Molecular Function	GO:0008948	oxaloacetate decarboxylase activity	1.61E-03	1.74E-01	1.74E-02	9.13E-02	1	4	ME1
GO: Molecular Function	GO:1902121	lithocholic acid binding	2.01E-03	2.17E-01	1.81E-02	9.51E-02	1	5	VDR
GO: Molecular Function	GO:0019834	phospholipase A2 inhibitor activity	2.01E-03	2.17E-01	1.81E-02	9.51E-02	1	5	ANXA1
GO: Molecular Function	GO:0005127	ciliary neurotrophic factor receptor binding	2.81E-03	3.03E-01	2.33E-02	1.23E-01	1	7	CLCF1
GO: Molecular Function	GO:0016615	malate dehydrogenase activity	3.21E-03	3.47E-01	2.48E-02	1.30E-01	1	8	ME1
GO: Molecular Function	GO:0061134	peptidase regulator activity	4.22E-03	4.55E-01	2.89E-02	1.52E-01	2	251	PI3,PRSS22
GO: Molecular Function	GO:1990814	DNA/DNA annealing activity	4.41E-03	4.77E-01	2.89E-02	1.52E-01	1	11	ANXA1
GO: Molecular Function	GO:0032052	bile acid binding	4.81E-03	5.20E-01	2.89E-02	1.52E-01	1	12	VDR
GO: Molecular Function	GO:0005499	vitamin D binding	4.81E-03	5.20E-01	2.89E-02	1.52E-01	1	12	VDR
GO: Molecular Function	GO:0004859	phospholipase inhibitor activity	5.21E-03	5.63E-01	2.96E-02	1.56E-01	1	13	ANXA1
GO: Molecular Function	GO:0140666	annealing activity	5.61E-03	6.06E-01	3.03E-02	1.60E-01	1	14	ANXA1
GO: Molecular Function	GO:0055102	lipase inhibitor activity	7.21E-03	7.79E-01	3.71E-02	1.95E-01	1	18	ANXA1
GO: Molecular Function	GO:0098641	cadherin binding involved in cell-cell adhesion	7.61E-03	8.22E-01	3.74E-02	1.97E-01	1	19	ANXA1
GO: Molecular Function	GO:0046965	nuclear retinoid X receptor binding	9.21E-03	9.94E-01	4.32E-02	2.28E-01	1	23	VDR
GO: Biological Process	GO:0018149	peptide cross-linking	8.69E-05	7.24E-02	3.06E-02	2.24E-01	2	37	PI3,ANXA1
GO: Biological Process	GO:0002828	regulation of type 2 immune response	9.17E-05	7.64E-02	3.06E-02	2.24E-01	2	38	CLCF1,ANXA1
GO: Biological Process	GO:0002285	lymphocyte activation involved in immune response	1.10E-04	9.19E-02	3.06E-02	2.24E-01	3	264	CD180,CLCF1,ANXA1
GO: Biological Process	GO:0042092	type 2 immune response	1.79E-04	1.49E-01	3.69E-02	2.69E-01	2	53	CLCF1,ANXA1
GO: Biological Process	GO:0046883	regulation of hormone secretion	2.99E-04	2.49E-01	3.69E-02	2.69E-01	3	370	CLCF1,ANXA1,VDR
GO: Biological Process	GO:0002366	leukocyte activation involved in immune response	3.16E-04	2.63E-01	3.69E-02	2.69E-01	3	377	CD180,CLCF1,ANXA1
GO: Biological Process	GO:0002263	cell activation involved in immune response	3.26E-04	2.71E-01	3.69E-02	2.69E-01	3	381	CD180,CLCF1,ANXA1
GO: Biological Process	GO:0046651	lymphocyte proliferation	4.31E-04	3.59E-01	3.69E-02	2.69E-01	3	419	CD180,CLCF1,ANXA1
GO: Biological Process	GO:0032943	mononuclear cell proliferation	4.52E-04	3.77E-01	3.69E-02	2.69E-01	3	426	CD180,CLCF1,ANXA1
GO: Biological Process	GO:0046879	hormone secretion	4.90E-04	4.08E-01	3.69E-02	2.69E-01	3	438	CLCF1,ANXA1,VDR
GO: Biological Process	GO:0009914	hormone transport	5.48E-04	4.57E-01	3.69E-02	2.69E-01	3	455	CLCF1,ANXA1,VDR
GO: Biological Process	GO:0070661	leukocyte proliferation	5.91E-04	4.93E-01	3.69E-02	2.69E-01	3	467	CD180,CLCF1,ANXA1
GO: Biological Process	GO:0002312	B cell activation involved in immune response	6.38E-04	5.31E-01	3.69E-02	2.69E-01	2	100	CD180,CLCF1
GO: Biological Process	GO:0051050	positive regulation of transport	6.58E-04	5.48E-01	3.69E-02	2.69E-01	4	1200	MTCL1,CLCF1,ANXA1,VDR
GO: Biological Process	GO:1903036	positive regulation of response to wounding	6.64E-04	5.53E-01	3.69E-02	2.69E-01	2	102	CLCF1,ANXA1
GO: Biological Process	GO:0042100	B cell proliferation	1.04E-03	8.68E-01	4.61E-02	3.37E-01	2	128	CD180,CLCF1
GO: Biological Process	GO:0010980	positive regulation of vitamin D 24-hydroxylase activity	1.16E-03	9.68E-01	4.61E-02	3.37E-01	1	3	VDR
GO: Biological Process	GO:0010979	regulation of vitamin D 24-hydroxylase activity	1.16E-03	9.68E-01	4.61E-02	3.37E-01	1	3	VDR
GO: Biological Process	GO:0032101	regulation of response to external stimulus	1.17E-03	9.73E-01	4.61E-02	3.37E-01	4	1396	CD180,CLCF1,ANXA1,VDR
GO: Biological Process	GO:1900138	negative regulation of phospholipase A2 activity	1.55E-03	1.00E+00	4.61E-02	3.37E-01	1	4	ANXA1
GO: Biological Process	GO:1900155	negative regulation of bone trabecula formation	1.55E-03	1.00E+00	4.61E-02	3.37E-01	1	4	VDR

GO: Biological Process	GO:1900154	regulation of bone trabecula formation	1.55E-03	1.00E+00	4.61E-02	3.37E-01	1	4	VDR
GO: Biological Process	GO:0048295	positive regulation of isotype switching to IgE isotypes	1.55E-03	1.00E+00	4.61E-02	3.37E-01	1	4	CLCF1
GO: Biological Process	GO:0051466	positive regulation of corticotropin-releasing hormone secretion	1.55E-03	1.00E+00	4.61E-02	3.37E-01	1	4	CLCF1
GO: Biological Process	GO:2000830	positive regulation of parathyroid hormone secretion	1.55E-03	1.00E+00	4.61E-02	3.37E-01	1	4	VDR
GO: Biological Process	GO:0014839	myoblast migration involved in skeletal muscle regeneration	1.55E-03	1.00E+00	4.61E-02	3.37E-01	1	4	ANXA1
GO: Biological Process	GO:0060057	apoptotic process involved in mammary gland involution	1.55E-03	1.00E+00	4.61E-02	3.37E-01	1	4	VDR
GO: Biological Process	GO:0060058	positive regulation of apoptotic process involved in mammary gland involution	1.55E-03	1.00E+00	4.61E-02	3.37E-01	1	4	VDR
GO: Biological Process	GO:0023061	signal release	1.80E-03	1.00E+00	4.62E-02	3.37E-01	3	685	CLCF1,ANXA1,VDR
GO: Biological Process	GO:2000828	regulation of parathyroid hormone secretion	1.94E-03	1.00E+00	4.62E-02	3.37E-01	1	5	VDR
GO: Biological Process	GO:0043397	regulation of corticotropin-releasing hormone secretion	1.94E-03	1.00E+00	4.62E-02	3.37E-01	1	5	CLCF1
GO: Biological Process	GO:0043396	corticotropin-releasing hormone secretion	1.94E-03	1.00E+00	4.62E-02	3.37E-01	1	5	CLCF1
GO: Biological Process	GO:0045629	negative regulation of T-helper 2 cell differentiation	1.94E-03	1.00E+00	4.62E-02	3.37E-01	1	5	ANXA1
GO: Biological Process	GO:0010817	regulation of hormone levels	2.11E-03	1.00E+00	4.62E-02	3.37E-01	3	724	CLCF1,ANXA1,VDR
GO: Biological Process	GO:0070564	positive regulation of vitamin D receptor signaling pathway	2.32E-03	1.00E+00	4.62E-02	3.37E-01	1	6	VDR
GO: Biological Process	GO:0002322	B cell proliferation involved in immune response	2.32E-03	1.00E+00	4.62E-02	3.37E-01	1	6	CD180
GO: Biological Process	GO:0033031	positive regulation of neutrophil apoptotic process	2.32E-03	1.00E+00	4.62E-02	3.37E-01	1	6	ANXA1
GO: Biological Process	GO:0030216	keratinocyte differentiation	2.52E-03	1.00E+00	4.62E-02	3.37E-01	2	200	ANXA1,VDR
GO: Biological Process	GO:0050671	positive regulation of lymphocyte proliferation	2.57E-03	1.00E+00	4.62E-02	3.37E-01	2	202	CLCF1,ANXA1
GO: Biological Process	GO:0032946	positive regulation of mononuclear cell proliferation	2.64E-03	1.00E+00	4.62E-02	3.37E-01	2	205	CLCF1,ANXA1
GO: Biological Process	GO:0071396	cellular response to lipid	2.69E-03	1.00E+00	4.62E-02	3.37E-01	3	788	CD180,ANXA1,VDR
GO: Biological Process	GO:1902339	positive regulation of apoptotic process involved in morphogenesis	2.71E-03	1.00E+00	4.62E-02	3.37E-01	1	7	VDR
GO: Biological Process	GO:0060558	regulation of calcidiol 1-monooxygenase activity	2.71E-03	1.00E+00	4.62E-02	3.37E-01	1	7	VDR
GO: Biological Process	GO:0048293	regulation of isotype switching to IgE isotypes	2.71E-03	1.00E+00	4.62E-02	3.37E-01	1	7	CLCF1
GO: Biological Process	GO:0048289	isotype switching to IgE isotypes	2.71E-03	1.00E+00	4.62E-02	3.37E-01	1	7	CLCF1
GO: Biological Process	GO:0060745	mammary gland branching involved in pregnancy	2.71E-03	1.00E+00	4.62E-02	3.37E-01	1	7	VDR
GO: Biological Process	GO:0097350	neutrophil clearance	2.71E-03	1.00E+00	4.62E-02	3.37E-01	1	7	ANXA1
GO: Biological Process	GO:0035898	parathyroid hormone secretion	2.71E-03	1.00E+00	4.62E-02	3.37E-01	1	7	VDR
GO: Biological Process	GO:0046887	positive regulation of hormone secretion	2.72E-03	1.00E+00	4.62E-02	3.37E-01	2	208	CLCF1,VDR
GO: Biological Process	GO:1903530	regulation of secretion by cell	2.94E-03	1.00E+00	4.82E-02	3.52E-01	3	813	CLCF1,ANXA1,VDR
GO: Biological Process	GO:1904747	positive regulation of apoptotic process involved in development	3.10E-03	1.00E+00	4.82E-02	3.52E-01	1	8	VDR
GO: Biological Process	GO:0070459	prolactin secretion	3.10E-03	1.00E+00	4.82E-02	3.52E-01	1	8	ANXA1
GO: Biological Process	GO:0070665	positive regulation of leukocyte proliferation	3.12E-03	1.00E+00	4.82E-02	3.52E-01	2	223	CLCF1,ANXA1
GO: Biological Process	GO:0050778	positive regulation of immune response	3.12E-03	1.00E+00	4.82E-02	3.52E-01	3	831	CD180,CLCF1,ANXA1
GO: Biological Process	GO:0002252	immune effector process	3.24E-03	1.00E+00	4.91E-02	3.59E-01	3	842	CD180,CLCF1,ANXA1
GO: Biological Process	GO:1903412	response to bile acid	3.48E-03	1.00E+00	4.92E-02	3.59E-01	1	9	VDR
GO: Biological Process	GO:0006108	malate metabolic process	3.48E-03	1.00E+00	4.92E-02	3.59E-01	1	9	ME1
GO: Biological Process	GO:0010519	negative regulation of phospholipase activity	3.48E-03	1.00E+00	4.92E-02	3.59E-01	1	9	ANXA1
GO: Biological Process	GO:0045627	positive regulation of T-helper 1 cell differentiation	3.48E-03	1.00E+00	4.92E-02	3.59E-01	1	9	ANXA1
GO: Biological Process	GO:1903034	regulation of response to wounding	3.57E-03	1.00E+00	4.95E-02	3.62E-01	2	239	CLCF1,ANXA1
GO: Cellular Component	GO:0001533	cornified envelope	2.88E-04	2.25E-02	1.99E-02	9.83E-02	2	68	PI3,ANXA1
GO: Cellular Component	GO:0016328	lateral plasma membrane	6.72E-04	5.25E-02	1.99E-02	9.83E-02	2	104	MTCL1,ANXA1
GO: Cellular Component	GO:0097059	CNTFR-CLCF1 complex	7.65E-04	5.97E-02	1.99E-02	9.83E-02	1	2	CLCF1
GO: Cellular Component	GO:0097058	CRLF-CLCF1 complex	1.15E-03	8.95E-02	2.09E-02	1.03E-01	1	3	CLCF1
GO: Cellular Component	GO:0031012	extracellular matrix	1.59E-03	1.24E-01	2.09E-02	1.03E-01	3	665	CD180,PI3,ANXA1
GO: Cellular Component	GO:0030312	external encapsulating structure	1.60E-03	1.25E-01	2.09E-02	1.03E-01	3	667	CD180,PI3,ANXA1
GO: Cellular Component	GO:0042383	sarcolemma	2.19E-03	1.71E-01	2.24E-02	1.11E-01	2	189	ANXA1,VDR
GO: Cellular Component	GO:0097427	microtubule bundle	2.29E-03	1.79E-01	2.24E-02	1.11E-01	1	6	MTCL1

GO: Cellular Component	GO:0001651	dense fibrillar component	2.68E-03	2.09E-01	2.32E-02	1.15E-01	1	7	VDR
GO: Cellular Component	GO:0042629	mast cell granule	3.06E-03	2.38E-01	2.38E-02	1.18E-01	1	8	ANXA1
GO: Cellular Component	GO:0031232	extrinsic component of external side of plasma membrane	4.96E-03	3.87E-01	3.52E-02	1.74E-01	1	13	ANXA1
